# Recent Progress in 3D Printing of Elastic and High-Strength Hydrogels for the Treatment of Osteochondral and Cartilage Diseases

**DOI:** 10.3389/fbioe.2020.604814

**Published:** 2020-11-27

**Authors:** Wenli Dai, Muyang Sun, Xi Leng, Xiaoqing Hu, Yingfang Ao

**Affiliations:** ^1^Beijing Key Laboratory of Sports Injuries, Institute of Sports Medicine, Peking University Third Hospital, Beijing, China; ^2^Medical Imaging Center, The First Affiliated Hospital of Guangzhou University of Chinese Medicine, Guangzhou, China

**Keywords:** 3D printing, hydrogel, elasticity, high strength, cartilage diseases osteochondral diseases

## Abstract

Despite considerable progress for the regenerative medicine, repair of full-thickness articular cartilage defects and osteochondral interface remains challenging. This low efficiency is largely due to the difficulties in recapitulating the stratified zonal architecture of articular cartilage and engineering complex gradients for bone-soft tissue interface. This has led to increased interest in three-dimensional (3D) printing technologies in the field of musculoskeletal tissue engineering. Printable and biocompatible hydrogels are attractive materials for 3D printing applications because they not only own high tunability and complexity, but also offer favorable biomimetic environments for live cells, such as porous structure, high water content, and bioactive molecule incorporation. However, conventional hydrogels are usually mechanically weak and brittle, which cannot reach the mechanical requirements for repair of articular cartilage defects and osteochondral interface. Therefore, the development of elastic and high-strength hydrogels for 3D printing in the repairment of cartilage defects and osteochondral interface is crucial. In this review, we summarized the recent progress in elastic and high-strength hydrogels for 3D printing and categorized them into six groups, namely ion bonds interactions, nanocomposites integrated in hydrogels, supramolecular guest–host interactions, hydrogen bonds interactions, dynamic covalent bonds interactions, and hydrophobic interactions. These 3D printed elastic and high-strength hydrogels may provide new insights for the treatment of osteochondral and cartilage diseases.

## Introduction

Damage of cartilage and osteochondral tissue is one of the most common health problems worldwide, which occurred due to various reasons such as disease, injuries, and trauma. Traumatic injuries to the joint, osteochondritis dissecans, and osteoarthritis are the most common reasons for osteochondral and cartilage diseases. Investigations by arthroscopy have reported the prevalence of cartilage and osteochondral damage was 60% in the general population (Liu et al., 2017c). As life expectancy is expected to longer in the next few coming decades, age-related musculoskeletal disorders such as osteoarthritis will become a major health concern in our societies, which constitutes one of the most relevant causes of incapacity in the elderly (Fellows et al., 2016; Richardson et al., 2016). Moreover, although osteoarthritis was previously believed to be a disease of the elderly, it develops much earlier than originally thought, and ranks among the top 20 in the 40–45 age group (Roos and Arden, 2016). It is foreseeable that it will cause a large economic burden for health systems around the world (Bijlsma et al., 2011; Rausch Osthoff et al., 2018).

**FIGURE 1:**
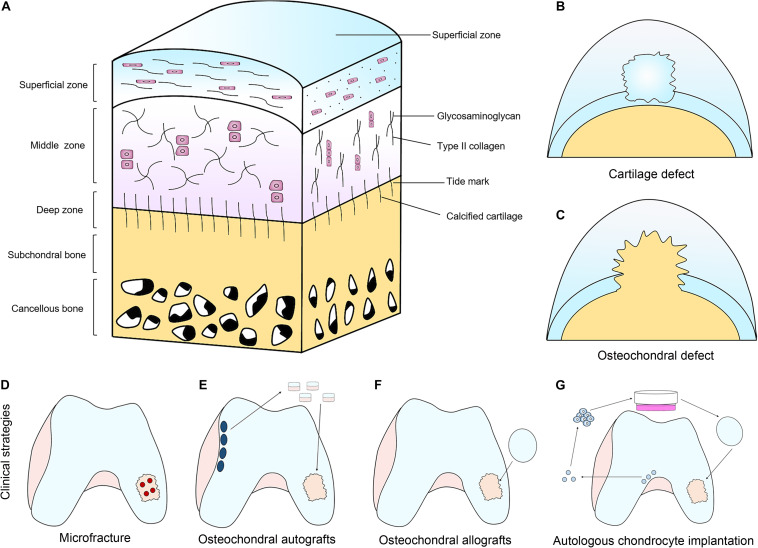
The structure of articular cartilage and clinical treatment strategies. **(A)** Articular cartilage consists of chondrocytes embedded in a defined structure of glycosaminoglycans and collagen fibers. **(B,C)** Two main types of defects can occur; chondral defects **(B)**, which only penetrate the cartilage and osteochondral defects **(C)**, which penetrate the subchondral bone. **(D–G)** Current clinical treatment strategies for full-thickness cartilage defects and osteochondral interface include microfracture **(D)**, osteochondral autografts **(E)** and allografts **(F)**, as well as autologous chondrocyte implantation **(G)**.

Unlike the majority of other tissues, cartilage is low in cellularity and basically avascular in nature (Figure 1A; Huey et al., 2012). Therefore, cartilage lacks the ability of self-healing due to the lack of proper progenitor cells and adequate nutrients. If the defect of cartilage is left untreated, it can cause irreversible and progressive deterioration of joints, leading to osteoarthritis, and eventually, disabilities (Chen et al., 2009). Current clinical treatment strategies for full-thickness cartilage defects (Figure 1B) and osteochondral interface (Figure 1C) include microfracture (Figure 1D; MacDonald et al., 2016; Polat et al., 2016), osteochondral autografts (Figure 1E) and allografts (Figure 1F; Benazzo et al., 2008; Haene et al., 2012), as well as autologous chondrocyte implantation (Figure 1G; Selmi et al., 2008; Harris et al., 2010; Gou et al., 2020).

Although they are often used clinically, significant drawbacks and limitations still exist. The microfractures drills small holes through the subchondral bone to allow the bone marrow to flow into the defect area. By introducing biomolecules and stem cells into the defect, it is expected to promote cartilage and osteochondral regeneration. However, it usually causes the fibrocartilage formation that has inferior properties compared with the hyaline cartilage (Galperin et al., 2013; Bert, 2015; Redondo et al., 2018). Autologous cartilage graft has been used clinically for decades to regenerate articular cartilage and has achieved satisfactory surgical results. However, it still has several limitations such as shortage of chondrocyte source, long chondrocyte harvesting time, difficulty of chondrocyte solution fixation (Tuan, 2007), as well as lack of effectiveness for aged patients (Giannoni et al., 2005). In addition, it is known that autologous cartilage graft is unable to repair full-thickness cartilage and osteochondral interface, which need to repair the subchondral bone at the same time. Allografts suffer from low cell viability due to graft storage, immunorejection, limited tissue supply, insufficient integration, as well as possibility of disease spread. Compared with allografts, autologous transplantation not only lacks tissue source and integration, but also requires additional surgery, which can cause morbidity in the donor site (Huey et al., 2012; Galperin et al., 2013).

To solve these problems, cartilage and osteochondral tissue engineering have been recommended to promote more effective treatments. One of the promise methods is to make artificial constructs to imitate the mechanical properties, biological functions, and architectural features of native cartilage and osteochondral tissue. Via combining advanced three-dimensional (3D) printing technology and specially designed biopolymers, it becomes possible to develop materials with sufficient mechanical properties to articular cartilage (Galperin et al., 2013). The printable materials used for cartilage and osteochondral tissue repair should have sufficient mechanical strength, strong interfacial strength, desirable biocompatibility, and adequate printability. The ideal inks are usually flowable fluids, which can be easily extruded and quickly solidified to maintain their shape using physical or chemical stimulations. They can also supply favorable environments that simulate extracellular matrix for different cells, such as porous structure, tunable mechanical properties, and high water content. Moreover, these inks can directly load bioactive molecules and cells (Tibbitt and Anseth, 2009; Ozbolat and Hospodiuk, 2016). Most inks are based on natural or synthetic polymers, such as gelatin (Van Hoorick et al., 2019), hyaluronic acid (Vega et al., 2017), alginate (Rastogi and Kandasubramanian, 2019), silk fibroin (Ribeiro et al., 2018), and poly(ethylene glycol) (Arcaute et al., 2006). However, traditional hydrogels are usually weak and brittle, making it difficult to mimic the mechanical properties of cartilage tissue, which has high strength, and is elastic and shock-absorbent (Pascual-Garrido et al., 2018). Therefore, it is promising to construct high-strength, elastic, and biomimetic hydrogels to mimic the mechanical properties of native articular cartilage osteochondral tissues.

Unlike the traditional hydrogels formed by covalent networks, which is hard to cause huge changes for the properties of hydrogels due to an unchanging covalent framework, hydrogels formed by non-covalent networks exhibit reversible crosslinking with a kinetic rate that can change with the environment. Non-covalent bonds can be broken to dissipate energy under stress, and self-healed to reform the hydrogels. The hydrogels can also significantly change over time without permanent changes to the hydrogel network (Sun et al., 2012). Due to these attractive properties, recently, non-covalently formed hydrogels have emerged as a neoteric class of scaffolds that integrate hydrogels with reversible crosslinking to develop advanced features including high-strength, elastic, and self-healing properties, which provide great advantages for cartilage and osteochondral tissue engineering.

In this review, we focus on recent progress in the development of high-strength and elastic hydrogels for cartilage and osteochondral tissue engineering. We review various classes of high-strength and elastic hydrogel systems including: (1) physical associations to assemble hydrogels (hydrogen bonds interactions, hydrophobic interactions, supramolecular guest–host interactions, and ionic bonds interactions), (2) physical associations to assemble particle-based hydrogels (nanocomposites integrated in hydrogels), and (3) dynamic covalent chemistry to form hydrogels (dynamic covalent bonds interactions). The various mechanisms of hydrogel formation, mechanical properties, and applications in cartilage and osteochondral regeneration are summarized for each subgroup. Additionally, recent advances in 3D bioprinting for cartilage and osteochondral tissue engineering are also discussed.

## Elastic and High-Strength Hydrogels

### Physical Associations to Assemble Hydrogels

#### Hydrogen Bonds Interactions

The hydrogen bonds are usually used to strengthen hydrogels due to its relative stability in aqueous condition. In addition, the dissociation energy of hydrogen bonds is relatively low. Moreover, the strength of multiple hydrogen bonds could be equivalent to a covalent bond (Wang W. et al., 2017). The hydrogen bonds occur in competition with water molecules in aqueous condition, whose contributions can be reduced by using multiple hydrogen bonding motifs with high dimerization affinity. These motifs can be used to make reversible physical network of a copolymer of a methacroyl monomer bearing a Ad-functionalized 2-ureido-4[1H]pyrimidinone (UPy) unit and N,N′-dimethylacrylamide (DMA) (Chirila et al., 2014) or a PEG chain bearing UPy moieties shielded from water by alkyl spacers (Dankers et al., 2012; Kieltyka et al., 2013) or apolar isophorone (Gemert et al., 2012). These hydrogels showed a reversible change from viscous fluids to stiff gels when cooling. Furthermore, the mechanical properties could be tuned by the addition of UPy-PEGs (Kieltyka et al., 2013). Notably, the hydrogels demonstrated a strong elastic property G′ of 18 kPa in angular frequency of 10 rad/s at 20°C.

The use of polymer chains with a series of hydrogen bonding sites can also generate strong hydrogen bond interactions in the hydrogel. Compared with simple amide hydrogen bond, dual amide hydrogen bonds are relatively more stable. Thus, study has designed a hydrogel based on N-acryloyl glycinamide (NAGA), a glycinamide-conjugated polymerizable monomer that consists of two amides (Dai et al., 2015). By photopolymerization of NAGA above 10 wt% concentration, this hydrogel could have excellent mechanical strength and a good fatigue resistance (Dai et al., 2015). These enhanced mechanical properties were attributed to the stable multiple hydrogen bonding domains acting as physical cross-links in the hydrogel. NAGA hydrogel also has self-healing property, remoldability, and thermoplasticity.

The strength of hydrogen bonds can also be significantly influenced by incorporating hydrophobic groups, which can further affect the mechanical properties of the hydrogels contain donor comonomer units and hydrogen bond acceptor. Study has reported introduction of methyl motif to acrylic acid (MAAc) can obviously increase the mechanical property of hydrogen-bonded hydrogels (Hu et al., 2015). The hydrogel based on 1-vinylimidazole and MAAc has a Young’s modulus up to 170 MPa (Ding et al., 2017; Zhang et al., 2018). By free-radical copolymerization of MAAc and N,N-dimethylacrylamide (DMAA) in an aqueous environment, study has prepared a hydrogel with a high Young’s modulus (28 MPa), fatigue resistance, stretch at break (800%), and tensile strength (2 MPa) (Hu et al., 2015). Study has also shown that the hydrogen bond donor carboxylic group of MAAc and strong hydrogen bond acceptor carbonyl group of DMAA could form multiple hydrogen bonds. By hydrophobic interactions of the α-methyl groups of MAAc units, it could further causing polymer-rich aggregates stabilized. These aggregates could act as sacrificial links to ensure energy dissipation in the hydrogels.

By adding hydrogen bonds interactions to the networks of hydrogels, studies have developed hydrogels with high strength for cartilage regeneration. In the study by Liu S. et al. (2020), a hydrogel scaffold was developed by thiol-ene Michael addition between DL-1,4-Dithiothreitol (DTT) and glycidyl methacrylate-modified poly (γ-glutamic acid) (γ-PGA-GMA) for cartilage regeneration. Sodium tetraborate decahydrate was introduced into the system to connect with DTT through hydrogen bond interaction and acted as catalyst for thiol-ene Michael addition to enhance the intensity of the hydrogel. The hydrogels could be compressed to 90% strain, with 0.95 MPa compression stresses. Moreover, cells cultured in the hydrogels showed good adhesion and proliferation abilities, and the hydrogels scaffolds with mesenchymal stem cells (MSCs) significantly enhanced the regeneration of cartilage in a rabbit model (Liu S. et al., 2020). In the study by Gao et al. (2019), a high-strength hydrogel composed of GelMA and cleavable poly(N-acryloyl 2-glycine) (PACG) is developed by photo-initiated polymerization. Introducing hydrogen bond-strengthened PACG causes a significant enhancement in the strengths of the hydrogel with a high compressive strength (12.4 MPa), outstanding tensile strength (1.1 MPa), large compression modulus (837 kPa), and high Young’s modulus (320 kPa) (Gao et al., 2019). Furthermore, the hydrogel not only supports cell adhesion and proliferation but also promotes gene expression of osteogenic-related and chondrogenic-related differentiation of MSCs. After 12 weeks of implantation, the hydrogel significantly promotes concurrent regeneration of cartilage and subchondral bone in a rat model (Gao et al., 2019).

#### Hydrophobic Interactions

Different from other non-covalent interactions that rely on direct intermolecular attraction, hydrophobic interactions are driven by the tendency of water molecules to maintain their hydrogen-bonded network intact around non-polar solutes. It can be altered by the presence of cosolutes, increased temperature, as well as size of species (Otto and Engberts, 2003). Polymer-based materials cross-linked by hydrophobic interactions can be prepared through bringing hydrophobic sequences inside or at the ends of hydrophilic chains (Winnik and Yekta, 1997). Due to the dynamic nature of the interactions, such non-covalent hydrogels is able to display exhibit self-healing and elastic capacity (Gulyuz and Okay, 2014).

Elastic and self-healing networks of hydrophobically cross-linked materials can be developed by micellar copolymerization of (1) hydrophilic comonomers, such as N-alkylacrylamides (N,N-dimethylacrylamide, N-isopropylacrylamide), acrylamide (AAm) or acrylic acid (AAc); with (2) large hydrophobic monomers, such as dococyl acrylate (C22) (Tuncaboylu et al., 2011), stearyl methacrylate (C18) (Tuncaboylu et al., 2011; Akay et al., 2013; Algi and Okay, 2014; Gulyuz and Okay, 2014); octylphenyl polyethoxyether acrylate (Jiang et al., 2010) in the presence of (3) a surfactant (cetyltrimethyl ammonium bromide CTAB, sodium dodecyl sulfate SDS); (4) salt (NaBr, NaCl). Micellar radical copolymerization is usually used to synthesize associative copolymers in an aqueous environment. However, long-chain alkyl(meth)acrylates have very low water-solubility, which was different from smaller hydrophobic N-alkyl(meth)acrylates or N-alkylacrylamides. Adding a sufficient amount of salt or co-surfactant will induce the morphological transformation of surfactant micelles. Larger micelles are able to compose of a large number of hydrophobes, and then they can grow further or adopt a different form to achieve thermodynamic feasibility (Tuncaboylu et al., 2011; Akay et al., 2013).

C22- and C18-acrylamide copolymer hydrogels with a SDS-NaCl system could reach an elastic modulus at 1 kPa. Study has demonstrated that after copolymerization, the hydrogel becomes mechanically stronger after the surfactant micelles are swollen and extracted, as the hydrophobic interaction is enhanced in the absence of surfactant (Tuncaboylu et al., 2011). After removing SDS micelles by equilibrium swelling in water, the tensile strength of a C18-acrylamide copolymer hydrogels could increase from 12 ± 1 to 78 ± 6 kPa, whereas the elongation at break change from 2,200% ± 350% to 650% ± 80% at the same time. At the same time, the hydrogels lost the self-heal ability due to longer times of hydrophobic associations. Additionally, the strength of hydrogels could also be increased via increasing the hydrophobic interactions or adding other covalent or physical crosslinking (Tuncaboylu et al., 2013), and balancing these two characteristics is crucial to optimize the mechanical properties of hydrogels.

By introducing hydrophobic interactions into the networks of hydrogels, studies have attempted to replicate the unique characteristics of cartilage in hydrogels. In the study by Means et al. (2019), a double network hydrogel, composed of poly(2-acrylamido-2-methylpropanesulfonic acid) (PAMPS) and poly(N-isopropylacrylamide-co-acrylamide) [P(NIPAAm-co-AAm)], was developed for cartilage replacement. In this hydrogel, PNIPAAm is used to achieve superior mechanical properties with its thermal transition temperature tuned above the physiological range. Compared with native cartilage, this hydrogel was confirmed to not only parallel the strength, modulus, and hydration of cartilage but also exhibit a 50% lower coefficient of friction (Means et al., 2019). The exceptional cartilage-like properties of the PAMPS/P(NIPAAm-co-AAm) hydrogels makes them candidates for synthetic cartilage grafts.

#### Supramolecular Guest–Host Interactions

In a supramolecular guest–host interaction, a guest moiety is physically inserted into another host moiety, and is held together by non-covalent bonds. Harada’s pioneering research proved that polyethylene oxide (PEO) can insert multiple α-cyclodextrin (CD) groups, which is the driving force for the research and development of supramolecular guest–host assembly hydrogels (Harada et al., 1992). Since then, various polymer structures with host–guest interactions were designed to develop supramolecular materials. Traditional covalent bonds cross-linked materials needed invasive surgical procedure to implant and promote drug delivery. To deal with this problem, supramolecular networks have been developed to make hydrogels injectable. Because of the non-covalent bonds in the network, studies have shown that the hydrogels had notably shear-thinning behavior to enable flow through an injector, and could reassemble at the injection site (Mann et al., 2017; Sahoo et al., 2018).

Self-healing and shear-thinning properties of supramolecular hydrogels have been demonstrated in different types of polymeric entities (Abdul Karim and Loh, 2015; Kai et al., 2015; Xue et al., 2018). Studies have found that the complexation with non-covalent interaction made the hydrogels reversible under shear cycles (Xue et al., 2018). Studies have also reported the sustained release properties for these materials (Ye et al., 2015; Abdul Karim et al., 2016). For example, study have revealed that PEO conjugated to tetraphenylethene (TPE) could cause aggregation-induced emission, and addition of α-CD could cause a 4–12-fold enhancement of fluorescence (Liow et al., 2017), study have also designed stimuli-responsive hydrogels with sol–gel phase based on the host–guest interactions (Yamaguchi et al., 2012; Qu et al., 2015). Azobenzene-based hydrogels were one of the most investigated materials because the trans-cis isomerization reaction based on visible light could cause the hydrogels being a sol–gel reversible phase (Zhao and Stoddart, 2009). Jiang et al. (2010) developed a polypseudorotaxane (PPR) material based on α-CDs and PEG. In this hydrogel, azobenzene derivative could act as competitive guest that cause trans-cis isomerization (Liao et al., 2010). Study has also constructed a photo-responsive supramolecular network including α-CD and dimethylamino-substituted azobenzene entity. In this hydrogel, trans-cis azobenzene transition occurred by the host–guest interaction between α-CD and azobenzene, which could lead to sol–gel phase (Wang J. et al., 2017).

**FIGURE 2:**
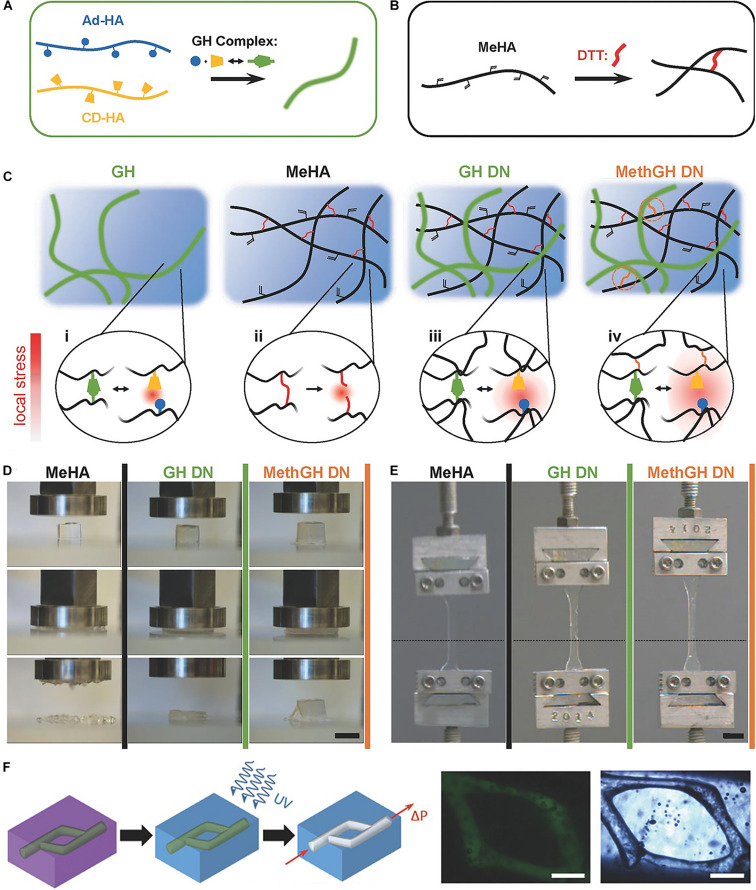
A tough and stretchable hydrogel by mixing the covalent crosslinking and host–guest pair of methacrylated hyaluronic acid. **(A)** Schematic of Ad and β-CD modified HA crosslinked through GH complexation. **(B)** Schematic of Michael addition crosslinking of MeHA by DTT. **(C)** Network architectures and local stress examined in a GH hydrogel, MeHA, GH DN, and MethGH DN. **(D)** Differing modes of compressive failure were observed between the hydrogels following compression to 90% strain. **(E)** Tensile testing of identically composed samples demonstrated a high degree of elasticity. **(F)** The printing of channels by writing an ink into a support gel that is modified for secondary crosslinking. Adapted with permission from Highley et al. (2015) and Rodell et al. (2016).

Via combining supramolecular and covalent crosslinking, hydrogels could be developed with extra strength. The pair of Adamantane guest and β-CD host has an equilibrium binding affinity of 3.2 × 10^4^ M^–1^. Hydrogel cross-linked only by the above host–guest interaction is relatively weak and deforms under stress (Chen and Jiang, 2011). Burdick et al. developed a tough and stretchable hydrogel by mixing the covalent crosslinking and host–guest pair of methacrylated hyaluronic acid (Figures 2A,B; Rodell et al., 2016). This hybrid supramolecular-covalent hydrogel has tunable mechanical properties, shear-thinning characteristic, as well as self-healing behavior, and can be used as bioink for 3D-printing (Figures 2C–F; Highley et al., 2015; Loebel et al., 2017). Cucurbitacin (CB) binded to cationic hydrophobic compounds has a strong equilibrium binding affinity. It can also accommodate two guest molecules and have a cross-linking effect (Barrow et al., 2015). When the guest is binded with CB and cross-linked by a part of covalent bonds, an interpenetrating network (IPN) hydrogel could be formed (Liu et al., 2017b). The hydrogel has high toughness and stretchability, and can extended up to 100 times its original length (Liu et al., 2017a).

**FIGURE 3:**
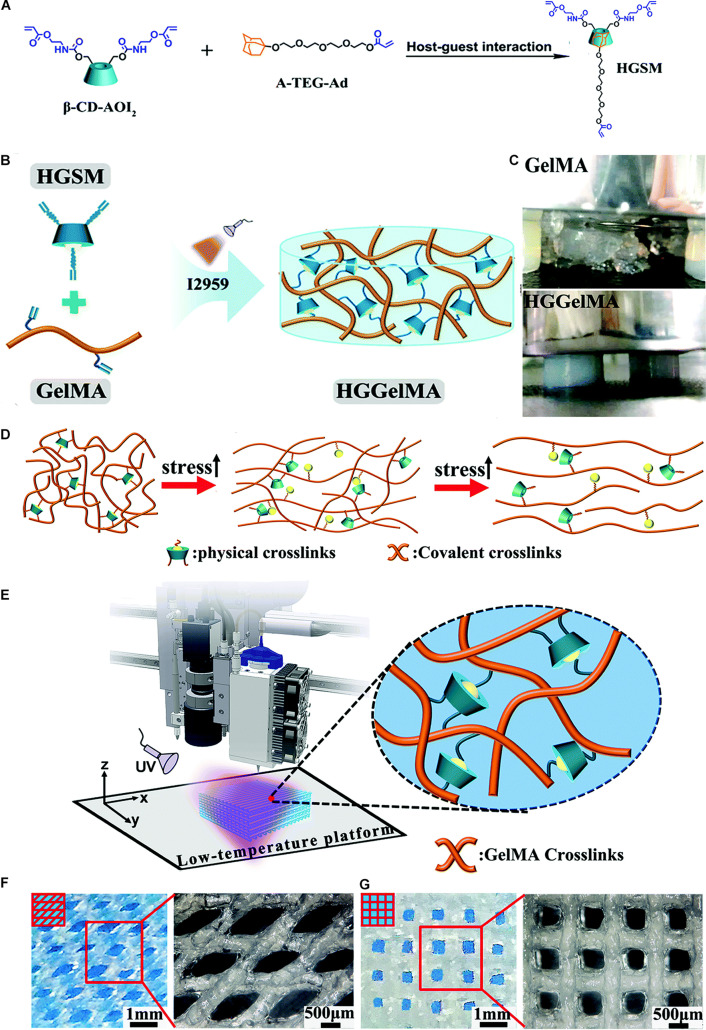
A novel hydrogel constructed with supramolecular guest–host interaction with three arms covalently crosslinked with GelMA. **(A)** Photographs showing the HGSM preparation process. **(B)** The design structure of the HGGelMA before and after crosslinking. **(C)** Digital images of the GelMA hydrogels that suffered the pressure of a 500 g weight and HGGelMA hydrogel that supported the pressure of a 1 kg weight. **(D)** Schematic of the mechanism involved in the robust and fatigue-resistant mechanical behavior of the HGGelMA. **(E)** 3D bioprinting of HGGelMA into scaffolds using HGGelMA precursors as a printing ink. **(F,G)** 3D rotational microscopy images showing the swelling equilibrium scaffolds under the swelling equilibrium when the scaffolds were constructed through layer-by-layer deposition with an alternating angle of 45° **(F)** or 90° **(G)** between adjacent layers and the resultant scaffolds. Adapted with permission from Wang Z. et al. (2019).

Recently, a novel hydrogel was constructed with supramolecular guest–host interaction with three arms covalently crosslinked with GelMA (Figure 3A; Wang Z. et al., 2019). This unique structure enabled the hydrogel to exhibit increased mechanical strength and show both 3D printing and self-healing properties. The three-armed supramolecular guest–host interaction was prepared through non-covalent host–guest interactions between isocyanatoethyl acrylate modified β-cyclodextrin (β-CD-AOI_2_) and acryloylated tetra-ethylene glycol-modified adamantane (A-TEG-Ad). Then, a host–guest supramolecular hydrogel (HGGelMA) was obtained via copolymerization between the arms of the guest–host interaction and GelMA to form a covalently crosslinked network (Figure 3B). The HGGelMA was robust, fatigue resistant, reproducible, and rapidly self-healing (Figure 3C). In the HGGelMA, the reversible non-covalent interactions could be re-established upon breaking, so as to heal the hydrogel and dissipate energy to prevent catastrophic fracture propagation (Figure 3D). Furthermore, the precursors of the HGGelMA were sufficiently viscous and could be rapidly photo crosslinked to produce a robust scaffold with an exquisite internal structure through 3D printing (Figures 3E,F; Wang Z. et al., 2019).

Enhanced osteochondral tissue regeneration could also be observed by using high-strength supramolecular hydrogels. In the study by Feng et al. (2016), supramolecular gelatin macromer was developed by host–guest interactions between photo-crosslinkable acrylated β-CD monomers and the aromatic residues of gelatin. The hydrogels are capable of sustaining excessive compressive and tensile strain. Moreover, by sustained release of kartogenin and TGF-β1, enhanced chondrogenesis of the encapsulated MSCs could be obtained *in vitro* and *in vivo* (Feng et al., 2016; Xu J. et al., 2019). Similarly, in the study by Wei et al. (2016), host–guest macromers are developed by molecular self-assembly between monoacrylated β-CD host monomers and adamantane-functionalized hyaluronic acid guest polymers. The hydrogels are capable of withstanding a compressive strain up to 80% and rapidly relaxing over 80% of the peak stress. Moreover, the hydrogels could not only sustain extended release of encapsulated TGF-β1 but also support chondrogenesis of the MSCs and promote cartilage regeneration in a rat model (Wei et al., 2016).

#### Ionic Bonds Interactions

As electrostatic interactions, the strength of ionic bonds interactions can change from a few carboxyl groups to the multidentate higher affinity metal ion coordination bonds. Although ionic bonds interactions are strong, charge shielding effects usually exists under physiological conditions. As a result, the metal ion coordination bonds with multidentate higher affinity and repeating charge unit are usually used to develop ionic hydrogel systems (McConnell et al., 2015; Winter and Schubert, 2016).

Hydrogel based on metal ion coordination bonds can be highly tunable. Hydrogel composing of imidazole can be crosslinked by various metal ions, such as Zn^2+^, Cu^2+^, or Co^2+^. Moreover, the number of metal ions and imidazole ligand can be tuned to convert the material from viscous liquids to tough gels. The strength of this hydrogel can be significantly enhanced by increasing the unbound imidazole. If the ratio of ligand/zinc is increased from 4.0 to 4.5, the tensile strength would decrease by 540%, whereas the extensibility would also increase by 600%. The crosslink exchange rate would increase via introducing a small part of unbound ligand, which would bring in the self-healing properties for the hydrogels. Therefore, in ionic bonds interactions, the unbound ligands have a critical impact in chain relaxation and stress distribution (Mozhdehi et al., 2016). Instead of using new metal ion-ligand pairs to adjust mechanical strength, two metal-ligand crosslinks can also exist in the same material, and the mechanical properties can be changed by adjusting the ratio of each metal ion-ligand pair (Grindy et al., 2015).

Ionic bonds can also be used as sacrificial bonds to achieve dissipation. Studies have made high-strength hydrogels by using ionically formed alginate-calcium and covalently formed polyacrylamide (Sun et al., 2012; Menyo et al., 2015). These materials have a fracture energy of around 9,000 Jm^–2^, and can extend to 20 times their original length (Sun et al., 2012; Menyo et al., 2015). They can also be modified with adhesive surfaces to develop super strong adhesives (Li J. et al., 2017). The poly(dimethylsiloxane) polymer chains can develop both weak and strong ligand binding interactions to Fe(III) through attaching the ligand 2,6-pyridinedicarboxamide. This can yield a highly stretchable hydrogel which has a fracture energy of 2,571 Jm, and can be stretched up to 45 times of the original length. In this material, the weaker carboxamide-iron bond can be ruptured under stress, and the polymer chain will gradually extend, whereas the pyridyl-iron bond ensures that the material can be still connected to iron under stress (Li C.H. et al., 2016).

**FIGURE 4:**
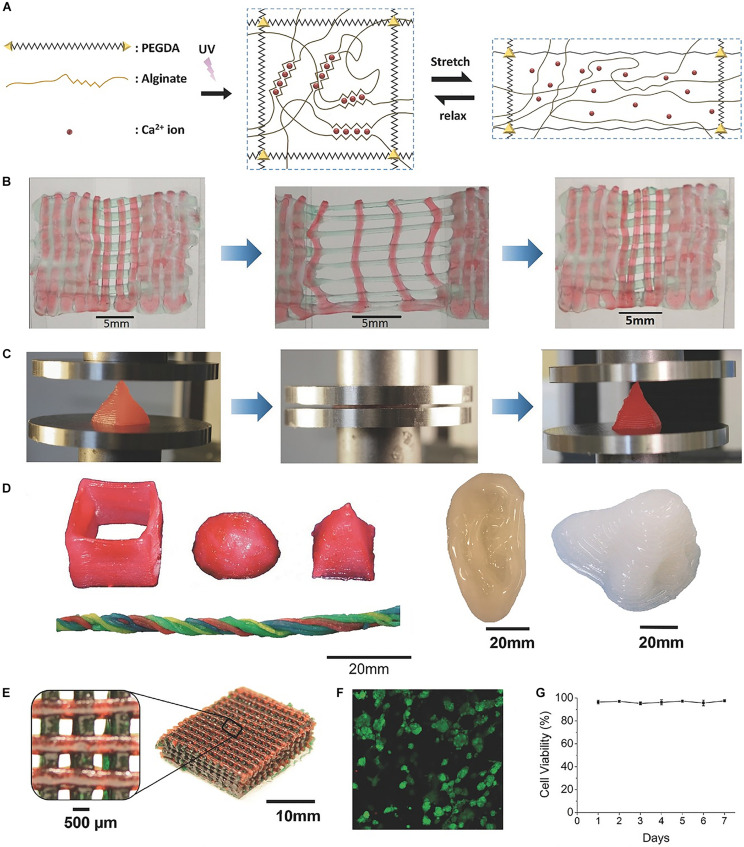
A highly stretchable and tough hydrogels developed by mixing sodium alginate and PEG to constitute an IPN. **(A)** Schematic diagrams of the biocompatible and tough hydrogel. **(B)** A printed bilayer mesh is uniaxially stretched to three times of its initial length. **(C)** A printed pyramid undergoes a compressive strain of 95% while returning to its original shape after relaxation. **(D)** Various 3D constructs printed with the hydrogel. **(E)** A mesh printed with the tough and biocompatible hydrogel. The mesh was used to host HEK cells. **(F)** Live-dead assay of HEK cells in a collagen hydrogel infused into the 3D printed mesh of the PEG–alginate–nanoclay hydrogel. **(G)** Viability of cells through 7 days. Adapted with permission from Hong et al. (2015a).

Similarly, highly stretchable and tough hydrogels can be also developed by combining ionic bonds interactions and covalent crosslinking. By mixing ionically crosslinked sodium alginate and covalently crosslinked poly (ethylene glycol) (PEG) to constitute an IPN (Figure 4; Hong et al., 2015a), study has reported a hydrogel with high fracture toughness and stretchability. In this hydrogel, incorporating reversible Ca^2+^ crosslinking into the hydrogels significantly increases their fracture energies. The increase in fracture energy is also accompanied by significant increase in stress–strain hysteresis, which suggests mechanical dissipation in the hydrogels under deformation. Additionally, because the longer polymer chains of PEG allow for higher stretchability of the hydrogel, the fracture energy of calcium-containing hydrogels increases significantly with the molecular weight of PEG. Moreover, by introducing the biocompatible nanoclay into the alginate-PEG hydrogel to control the viscosity of the pre-gel solution, shear-thinning properties of the hydrogel could be significantly enhanced, and it could be printed into diverse shapes such as twisted bundle, pyramid, hemisphere, as well as physiologically relevant shapes such as human ear models (Hong et al., 2015a).

The tunable stress relaxation properties of these ionic hydrogels made them applicable to tissue engineering. Study has revealed that compared with the hydrogel with the same gel modulus, cells are more widely distributed on the stress-relaxed soft viscoelastic hydrogels (Chaudhuri et al., 2015). This phenomenon was further explored by using alginate-calcium hydrogels with stress relaxation properties adjusted by different alginate molecular weights. As the stress relaxation rate increases, the cell proliferation and spreading increase, and the osteogenic differentiation of MSCs can also be enhanced (Chaudhuri et al., 2016). Hydrogels crosslinked by ionic bonds interactions could also be sufficiently robust for cartilage regeneration, which was examined on rabbit model bearing articular osteochondral defect. Through the generation of ionic interaction and borate bonding, poly(vinyl alcohol) (PVA) was crosslinked by 4-carboxyphenylboronic acid (CPBA) to bridge the polymer chains with the presence of calcium ions (Zhao et al., 2018). The dynamic gathering of CPBA could lead to a self-reinforcing effect inside the hydrogel matrix, resulting in high compressive and tensile moduli of the hydrogel over 1.0 MPa, including the highest compressive modulus up to 5.6 MPa. Moreover, after 3 months of implantation of this hydrogel into osteochondral defect in a rabbit model, smooth and complete cartilage layer was obtained, and the enrichment of glycosaminoglycan (GAG) and type-II collagen were observed in the cartilage layer (Zhao et al., 2018).

### Physical Associations to Assemble Particle-Based Hydrogels

#### Nanocomposites Integrated in Hydrogels

Hydrogels integrated with nanocomposite are defined as hydrated networks which are chemically or physically cross-linked by nanoparticles (Gaharwar et al., 2014). Different types of nanoparticles, including inorganic/ceramic nanoparticles, carbon-based nanomaterials, metal/metal oxide nanoparticles, and polymeric nanoparticles, can be mixed into the polymeric network to construct nanocomposite hydrogels (Gaharwar et al., 2014). Theoretically, the nanoscale composites have the greater surface area to volume ratios, which can not only increase the surface reactivity, but also improve the mechanical properties and bioavailability of the hydrogels. Additionally, since they can easily penetrate into focal tissues through narrow capillaries or epithelial inner layers, the efficacy of loaded therapeutic or bioactive agents can be improved (Pelgrift and Friedman, 2013; Jayaraman et al., 2015; Chen et al., 2018).

**FIGURE 5:**
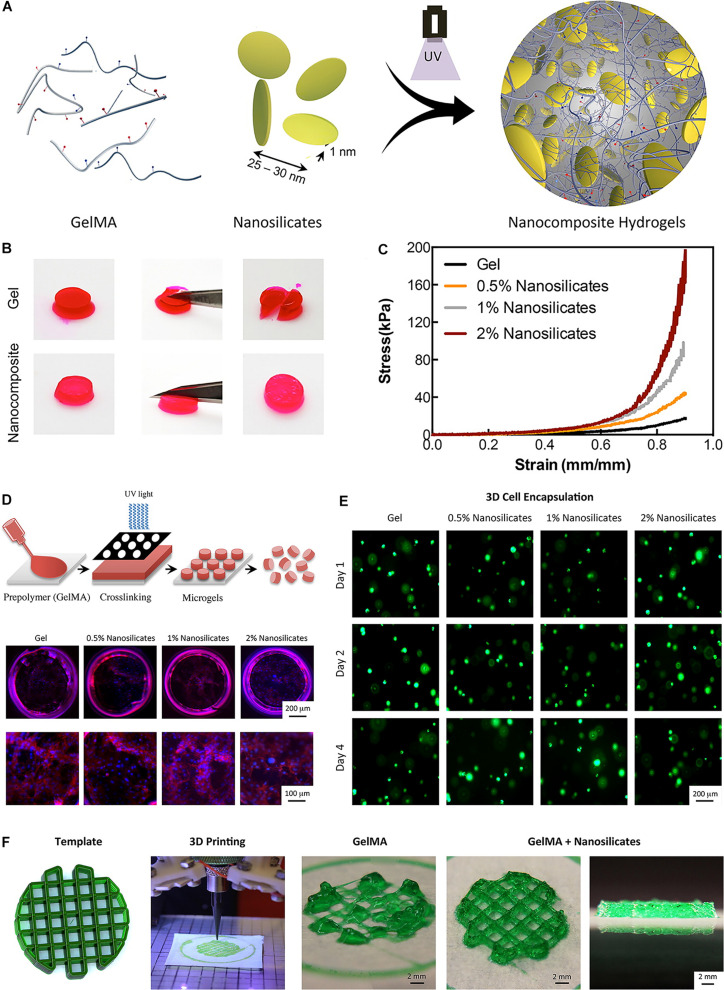
A bioactive nanoengineered hydrogels for tissue engineering. **(A)** Schematic representation of fabrication of nanocomposite hydrogels. **(B)** Optical images showing mechanical toughness of the Gel and nanocomposite hydrogels after deformation. **(C)** Nanocomposite hydrogels were subjected to unconfined compression up to 0.90 strain. **(D)** Schematic representation of fabrication of nanosilicate-loaded microgels. **(E)** In the 3D encapsulation of cells in nanocomposite hydrogels, all scaffolds supported cellular viability. **(F)** The addition of nanosilicate to GelMA results in a shear-thinning characteristic and can be printed to design complex structures. Adapted with permission from Xavier et al. (2015).

Some nanoparticles-reinforced hydrogels can be also used for 3D printing (Skardal et al., 2010; Xavier et al., 2015; Leppiniemi et al., 2017). Compared with the methacrylated gelatin (GelMA) hydrogel alone, a hydrogel mixed GelMA with nanosilicate showed a fourfold increase in compressive modulus (Figure 5A; Xavier et al., 2015). Moreover, a tenfold enhancement in compressive modulus could be obtained if the levels of nanosilicate increased to 2% (Figures 5B,C; Xavier et al., 2015). This hydrogel can be 3D printed to a precisely designed scaffold, and support the viability of encapsulated cells in 4 days of culture (Figures 5D–F). Castro et al. (2015) synthesized a nanomaterial consisting of osteoconductive nanocrystalline hydroxyapatite (nHA) and core-shell poly(lactic-co-glycolic) acid (PLGA) nanoparticles encapsulated with chondrogenic transforming growth-factor β1 (TGF-β1) for sustained delivery. As the primary inorganic component of bone, the hydroxyapatite nanocrystals could provide osteoconductivity, nanotexturization, and mechanical reinforcement (Castro et al., 2015). In this 3D printed osteochondral hydrogel, MSCs adhesion, proliferation, and osteochondral differentiation could be considerably increased *in vitro*. In the study by Palaganas et al. (2017), cellulose nanocrystal (CNC) from abaca plant is incorporated with Poly(ethylene glycol) diacrylate (PEGDA) to provide desirable strength for 3D printable biopolymer. Compared with the PEG hydrogel alone, the addition of 0.3 wt% CNC to the material would cause a twofold enhancement of tensile strength and a fourfold enhancement of fracture energy.

Several nanocomposite hydrogels have been developed to regenerate the extracellular matrix of cartilage. For example, Laponite-incorporated hydrogels are currently being investigated for their use as promising growth-factor-free solutions for osteochondral and cartilage regeneration. Study has reported that PEG incorporated with Laponite not only promoted cytoskeletal alignment of F-actin proteins and adhesion of cells, but also enhanced the proliferation and osteogenic differentiation of preosteoblast cells (Gaharwar et al., 2011; Xavier et al., 2015). Moreover, study has also observed the up-regulation of type-II collagen and proteoglycan in MSCs encapsulated within Laponite-based microcylinders (Dawson et al., 2011). This discovery further strengthens the notion that Laponite can stimulate both bone and cartilage differentiation of MSCs. This is a promising function that can be used to develop graded scaffolds for osteochondral regeneration. Study has also investigated a hybrid IPN mixed with silated hydroxylpropylmethyl cellulose and Laponite, which enhanced the mechanical properties of hydrogel without compromising the cytocompatibility, oxygen diffusion capability, generation of extracellular matrix components, and self-organization of chondrogenic cells (Boyer et al., 2018). Radhakrishnan et al. (2018) synthesized a semiinterpenetrating network hydrogel scaffold formed by nanohydroxyapatite and chondroitin sulfate nanoparticles, and used in subchondral and chondral hydrogel layers, respectively. By using this hybrid hydrogel, the regeneration of cartilage and subchondral bone was enhanced (Radhakrishnan et al., 2018). Zhang et al. (2015) designed a hybrid hydrogel, including magnetic nanoparticles, hyaluronic acid, type-II collagen, and PEG for regeneration of cartilage. The hydrogel displayed similar microstructure and chemical properties to that of hyaline cartilage, and has cell compatibility with MSCs *in vitro*. Notably, an external magnet could be used to direct this hydrogel remotely to the cartilage defect site (Zhang et al., 2015).

### Dynamic Covalent Chemistry to Form Hydrogels

#### Dynamic Covalent Bonds Interactions

In traditional hydrogels, the polymer network cross-linked by covalent bonds is irreversible, and is often too brittle that could easily fatigue. Reversible dynamical covalent interactions, such as imine bonds (Ying et al., 2014; Haldar et al., 2015; Xu Y. et al., 2018), disulfide bonds (Canadell et al., 2011; Deng et al., 2012; Li S. et al., 2016), phenylboronate complexations (Appel et al., 2015; Yesilyurt et al., 2016), acrylhydrazone bonds (Yu et al., 2015; Guo et al., 2017), Diels-Alder reactions (Liu and Chuo, 2013; Baker et al., 2017), and reversible radical reactions (Amamoto et al., 2012; Thi et al., 2020), are attractive strategies to develop elastic and high-strength hydrogels.

Combination of phenylboronic acid (PBA) and diols can develop a reversible boronate ester in aqueous environment, and can be brought into polymer network to form elastic materials. For instance, study has reported an elastic material using the reversible boronate ester complexation between salicylhydroxamic acid (SHA) and PBA. This material is able to exhibit various mechanical properties in a wide range of pH (Roberts et al., 2010). Study has also constructed elastic and self-healing hydrogels by combination of a catechol derived tetra-arm PEG (cPEG) with 1,3-benzenediboronic acid (BDBA) in phosphate buffer saline under alkaline pH at 20°C (He et al., 2011). Using PBA to replace benzoxaborole group would cause polymer network constructed at neutral pH (Dowlut and Hall, 2006).

Another major approach for developing dynamic covalent elastic and self-healing hydrogels is through imine bonds interactions, sometimes also called Schiff bases. For example, a self-healing hydrogel was made at room temperature by synthesizing telechelic dibenzaldehyde-terminated PEG and mixing it with an amine-containing biomacromolecule solution, such as peptides, chitosan, and gelatin (Zhang et al., 2011). Compared with the aliphatic Schiff base, the aromatic Schiff base is usually preferred because of its higher stability and can maintain the mechanical properties of the hydrogel (Engel et al., 1985).

**FIGURE 6:**
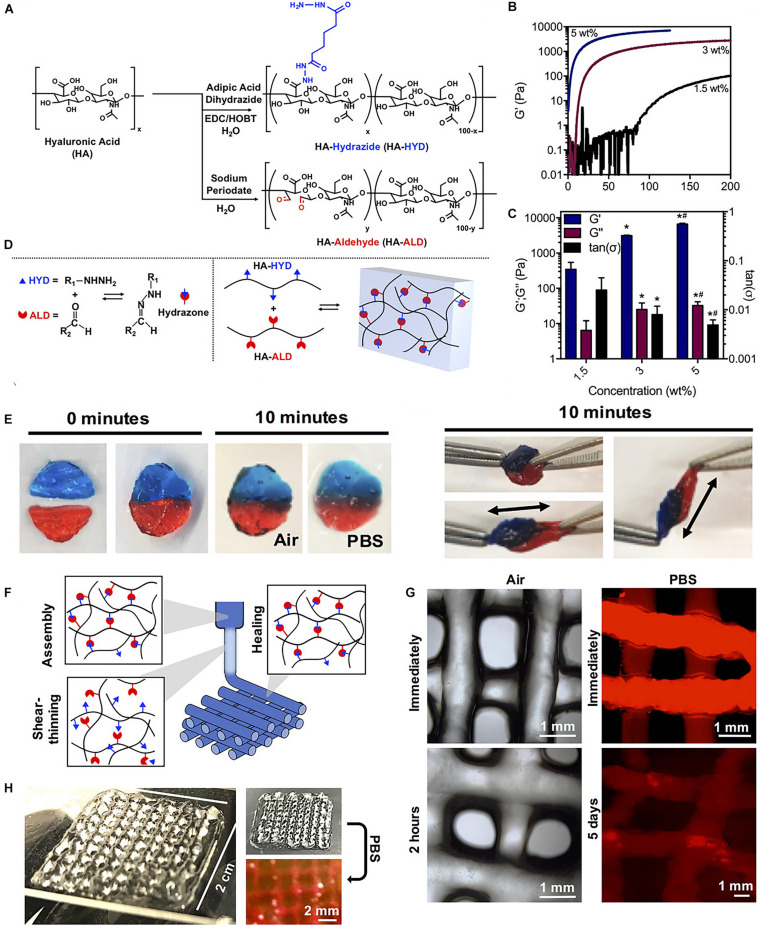
A tough and self-healing hydrogel by the dynamic covalent bonds interactions. **(A)** Synthesis schematic of HA-HYD and HA-ALD. **(B,C)** Time sweeps (1 Hz, 0.5% strain) and quantification of storage modulus (G′), loss modulus (G″), and tan(δ) of 1.5, 3, and 5 wt% hydrogels on shear-oscillatory rheometry. *p < 0.05 compared to 1.5 wt%, ^#^*p* < 0.05 compared to 3 wt%. **(D)** Schematic of the mechanism involved in the robust and fatigue-resistant mechanical behavior of the HGGelMA. **(E)** Self-healing of two dyed hydrogel discs in air and PBS, and manual stretching of healed hydrogel discs after 10 min. **(F)** Schematic of shear-thinning and self-healing of hydrogels during printing. **(G)** Photos of 4-layer lattices in air and in PBS. **(H)** Images of lattices in air and in PBS. Adapted with permission from Wang L.L. et al. (2018).

Study has also reported a shear-thinning and self-healing hydrogel crosslinked through dynamic covalent chemistry for 3D printing (Wang L.L. et al., 2018). Specifically, hyaluronic acid was modified with either hydrazide or aldehyde groups and mixed to form hydrogels containing a dynamic hydrazone bond (Figures 6A–D). Due to their shear-thinning and self-healing properties, the hydrogels could be extruded for 3D printing of structures with high shape fidelity, stability to relaxation, and cytocompatibility with encapsulated fibroblasts (> 80% viability) (Figures 6E–H). To increase the hydrogel strength, a second photocrosslinkable IPN was included that was used for orthogonal photostiffening and photopatterning through a thiol-ene reaction. Photostiffening increased the scaffold’s modulus (∼300%) while significantly decreasing erosion (∼70%), whereas photopatterning allowed for spatial modification of scaffolds with dyes (Wang L.L. et al., 2018).

Hydrogels crosslinked by dynamic covalent interactions have also used to enhance osteochondral tissue regeneration. In the study by Zhang et al. (2016), a phototriggered-imine-crosslinked hydrogel composed of hyaluronic acid, gelatin, and hydroxyapatite nanoparticles was prepared. In this hydrogel, o-nitrobenzyl alcohol moieties-modified hyaluronic acids (HA-NB) generate aldehyde groups under UV irradiation and then react with primary amino-bearing macromolecules on tissue surfaces, which is in favor of seamless tissue attachment and integration. Moreover, by integrating stem cell-derived exosomes (Liu et al., 2017d) or platelet-rich plasma (Liu et al., 2017e) into the hydrogel, it could enhance the proliferation and migration of MSCs and chondrocytes. After 12 weeks of implantation into a full-thickness cartilage defect in a rabbit model, the hydrogel could achieve integrative hyaline cartilage regeneration.

## 3D Bioprinting for Cartilage and Osteochondral Tissue Engineering

Since bio-inks are usually based on hydrogels, they are usually weak and therefore cannot mimic the mechanical properties of cartilage tissue, which has high strength, and is elastic and shock-absorbent. By co-printing with artificial thermoplastic polymers, significant enhancement of mechanical property can be obtained (Visser et al., 2015; Daly et al., 2016). However, study has reported that the reinforced hydrogels was unable to integrate at the boundary under physiological loading (Boere et al., 2014). Another method to develop mechanically reinforced hydrogels is to include IPN in hydrogels. By meticulously designing the primary and secondary networks, and combining them with appropriate proportion, the entanglement and energy dissipation of the IPN can be achieved, and it is possible to develop extremely high-strength and elastic materials comparable to native cartilage (Gong, 2010; Chen et al., 2015; Hong et al., 2015a). These methods may find utility for cartilage tissue bioprinting in the future.

Despite the significant progress in 3D printing technology, there are still many challenges for 3D bioprinting with living cells. One of the challenges is to maintain the viability of cells in the bio-inks during and after 3D bioprinting (Hölzl et al., 2016). Therefore, the cytocompatibility and bioactivity are two crucial features for bio-inks (Gopinathan and Noh, 2018). With regard to cytocompatibility, the bio-inks as well as the degradation molecules need to be non-toxic for cells *in vitro*, and not lead to the immune response *in vivo*. Several elastic and high-strength hydrogels have been reported with good cytocompatibility (Shin et al., 2013; Hong et al., 2015b; Paul et al., 2016). During the 3D bioprinting, shear stress, which can also cause cell damage, is thought as another risk factor for the reduction of the cells viability (Ning and Chen, 2017). Several factors can lead to an increase in shear stress, including decreasing the printing nozzle diameter, increasing extrusion speed, and increasing ink viscosity, which would result in cell damage during 3D bioprinting. However, inks with a higher viscosity are usually associated with better printing fidelity, and a smaller nozzle diameter can improve printing resolution. Both of the factors can help improve the printing quality. Therefore, there is a balance between the printability of bio-inks and viability of cells. By optimizing the properties of inks, and adjusting the printing parameters, studies have achieved both good cell viability and printability (Chang et al., 2008; Nair et al., 2009; Chung et al., 2013; Zhao et al., 2015; Ouyang et al., 2016). These studies further verified the relationship between the cell viability and the viscosity of the bio-inks. Therefore, the shear-thinning behavior is an important property for inks during bioprinting. As discussed above, the non-covalent interactions not only enhance the mechanical properties of the hydrogels, but also increase shear fluidization, which leads to the improvement of printability. The methods for crosslinking can also affect cell survival during 3D bioprinting. For example, light irradiation and photo-initiators can cause cell damage during 3D bioprinting. Therefore, choosing safe light wavelengths and biocompatible chemicals, as well as shortening the irradiation time would increase the cell viability. Moreover, incorporating bioactive molecules into the bio-inks is also a good way to increase cell viability for 3D bioprinting (Castro et al., 2015; Lin et al., 2016; Leppiniemi et al., 2017).

Although several elastic and high-strength inks have been developed for 3D printing and can culture with cells (Castro et al., 2015; Hong et al., 2015a), only a few inks were printed directly with living cells (Li H. et al., 2017; Xu C. et al., 2018). In the study by Li H. et al. (2017), an alginate/methylcellulose ink was printed directly with living cells and showed a high cell survival after 5 days of culture. Similarly, in the study by Xu C. et al. (2018), a PEG-PCL-DA hydrogel was developed to directly print with different living cells, and exhibited high cell viability with more than 80% cell survival after 7 days of culture. Several 3D printed elastic and high-strength hydrogels, such as collagen/hydroxyapatite and NAGA/nanoclay have been developed for bone tissue regeneration in animal models (Lin et al., 2016; Zhai et al., 2017). However, the application of 3D bioprinting of high-strength and elastic hydrogels with living cells is rarely reported for cartilage regeneration in animal models. Although the non-covalently formed hydrogels are promising inks for 3D bioprinting due to their shear-thinning property, these elastic hydrogels need to be further evaluated for 3D bioprinting and cartilage regeneration.

It is well known that cartilage is an anisotropic tissue, and the organization and composition varies in depth. However, there is a lack of studies that investigate the possibility of printing biological factor gradients to reconstruct the characteristics of cartilage and subchondral bone. During cartilage development, homeostasis, and repair, chondrocytes will experience gradients of physical and chemical cues, which can in turn affect cellular behaviors, such as proliferation, differentiation, and migration. During cartilage tissue engineering, biochemical and physical gradients can be added into the hydrogel. Study has demonstrated that an graded presentation of insulin-like growth factor (IGF) and recombinant human bone morphogenic protein 2 (BMP) can lead to localized chondrogenic and osteogenic differentiation of MSCs in hydrogels (Wang X. et al., 2009). Moreover, study has also reported that an graded presentation of TGF-β can cause the formation of highly heterogeneous cartilage tissues in hydrogels (Albro et al., 2016).

Increasing studies have highlighted the advantages of adding physical and biochemical gradients in osteochondral and cartilage tissue engineering. With combinations of biochemical and physical gradients, it could be possible to develop more native like cartilage and osteochondral tissues. In the study by Jeon et al. (2013), a combination of biochemical and physical gradients, including growth factor concentration, substrate stiffness, and RGD ligand presentation, was formed by using a dual syringe system to conduct the differentiation of MSCs toward osteogenic and chondrogenic lineages. Additionally, another study has also demonstrated how combinations of nanofiber alignments, substrate stiffness values, and growth factors could be used to develop the zone specific differentiation of MSCs (Moeinzadeh et al., 2016). Future development of bioprinting technology may make it possible to present a variety of gradual changes in chemical and physical cues over multiple length scales.

An alternative approach to construct stratified cartilage and osteochondral tissues is to bioprint gradients of different cells. The cell sources most widely used for cartilage and osteochondral tissue engineering are chondrocytes and MSCs (Leijten et al., 2013). Co-culture systems containing different cell sources could be combined in graded ratios to develop more stratified cartilage and osteochondral tissues (Hendriks et al., 2007; Bian et al., 2011; Leijten et al., 2013). Alternatively, zone-specific chondrocytes can be separated and bioprinted in layers to mimic the depth-dependent feature of osteochondral and cartilage tissue (Schuurman et al., 2016).

## Conclusion and Perspectives

Osteochondral damage is a very common clinical disease. Although there are several clinical treatment strategies for this disease, such as microfracture, osteochondral autografts and allografts, as well as autologous chondrocyte implantation, significant drawbacks and limitations still exist. As current surgical techniques for cartilage pathologies are insufficient to cure the cartilage injuries, and halt the development and progression of osteoarthritis, which has accelerated the development of alternative tissue engineering strategies. However, although cartilage is perceived as a simple tissue, developing biomaterials that can reach the mechanical properties of native cartilage remains a challenge.

Traditionally, the covalently bonded hydrogels are stiff and brittle, and cannot simulate mechanical properties of natural cartilage and osteochondral tissue, which has high strength, and is elastic and shock-absorbent (Pascual-Garrido et al., 2018). Unlike the covalently bonded hydrogels, non-covalently formed hydrogels have several remarkable properties. These hydrogels have self-healing property after remove of stress. They also have stress relaxation property that can promote cell proliferation and spreading. Moreover, they have tunable mechanical strength and shear-thinning property, which can convert from a viscous liquid to a stiff gel. By elaborately adjusting the non-covalent and covalent interactions in the networks, these tough and elastic hydrogels should have great potential for cartilage and osteochondral tissue regeneration (Table 1).

**TABLE 1:** Representative examples of elastic and high-strength hydrogels and applications for osteochondral and cartilage regeneration.

Interactions	Materials	Advantages	Application	Effect	References
Hydrogen bonds interactions	γ-PGA-GMA, DTT, and Sodium tetraborate decahydrate	The hydrogels can be compressed to nearly a 90% strain, with 0.95 MPa compression stresses	Cartilage defects in rabbit	Cells cultured in hydrogels exhibit good proliferation and adhesion abilities and the hydrogels scaffolds contained MSC enhance the regeneration of cartilage	Liu S. et al., 2020
	PACG and GelMA	The hydrogels have a high tensile strength (1.1 MPa), outstanding compressive strength (12.4 MPa), large Young’s modulus (320 kPa), and high compression modulus (837 kPa)	Osteochondral defects in rat	The hydrogel significantly facilitates concurrent regeneration of cartilage and subchondral bone	Gao et al., 2019
Hydrophobic interactions	PAMPS and P(NIPAAm-co-AAm)	The hydrogels demonstrate a high compressive strength (25 MPa), cartilage-like modulus (1 MPa), hydration (80%), and exhibit a 50% lower coefficient of friction than that of native articular cartilage	–	–	Means et al., 2019
Supramolecular guest–host interactions	Acrylated β-CD and gelatin	The hydrogels have a good extensibility (400%), and are fatigue resistant under repeated tensile loading–unloading cycles	Osteochondral defects in rat	The hydrogel can promote the regeneration of both hyaline cartilage and subchondral bone	Xu J. et al., 2019
	Acrylated β-CD and Ad-functionalized hyaluronic acid	The hydrogels are capable of withstanding a compressive strain up to at least 80% and rapidly relaxing over 80% of the peak stress	Cartilage defects in rat	The hydrogels not only sustain extended release of encapsulated TGF-β1 but also support chondrogenesis of the human MSCs and promote cartilage regeneration	Wei et al., 2016
Ionic bonds interactions	PVA and CPBA	The hydrogels are ultra-tough, showing maximum tensile strain, tensile and compressive fracture energies up to 1,600%, 600 and 25 kJ m^–2^, respectively	Osteochondral defects in rabbit	The hydrogels can promote smooth and complete cartilage regeneration	Zhao et al., 2018
Nanocomposites integrated in hydrogels	Silated hydroxypropylmethyl cellulose with Laponites	The increase of Laponites amount in hydrogel allows the modulus to reach a fourfold increase for 5% Laponites	Subcutaneous pockets of nude mice	Formation of a cartilage-like tissue with an ECM containing GAG and collagens is observed at 6 weeks implantation	Boyer et al., 2018
	Collagen and alginate with hydroxyapatite nanocrystals	Hydrogels with hydroxyapatite nanocrystals exhibit the highest modulus among all of the collagen-based hydrogels	–	The hydrogels can promote cell proliferation and upregulated hyaline cartilage markers *in vitro*	Zheng et al., 2014
	PEG-1000 and Pluronic F-127 copolymer with calcium phosphate nanocrystals	The hydrogels exhibit a good combination of compressive modulus (0.64 MPa) and tensile modulus (0.9 MPa). They can also bond well to native cartilage	–	–	Schlichting et al., 2011
Dynamic covalent bonds interactions	Hydrazine-modified elastin-like protein and aldehyde-modified hyaluronic acid	By tuning the ratio of aldehyde groups to hydrazine groups, hydrogels with variable hyaluronic acid concentration can be fabricated with comparable stiffness	–	The hydrogels can promote cartilage-marker gene expression and enhanced GAG deposition while minimize undesirable fibrocartilage phenotype in *in vitro*	Zhu et al., 2017

Despite the obvious advantages and significant progress, the ultimate aim of cartilage and osteochondral tissue engineering is to translate a promising therapeutic strategy to the clinics to regenerate and restore the articular cartilage for patients. Non-covalently formed hydrogels have a promising place among the biomaterials evaluated for clinical regeneration of cartilage and osteochondral tissues for patients with osteoarthritis due to their tunable mechanical strength and self-healing property. Moreover, because of their shear-thinning property, these hydrogels are promising inks for 3D bioprinting, which could potentially contribute to the next generation personalized strategy that addresses each patients’ requirement as it is a automated, computerized, and rapid technology to develop constructs that mimic native cartilage and osteochondral tissues. However, before translating to clinics, meticulous choice of these hydrogels with robust features such as regenerative potential, biocompatibility, and degradability assessed in pre-clinical trials is required.

## Author Contributions

WD and MS drafted the manuscript. XL, XH, and WD collected and sorted the information. YA and XH designed the conception and revised the manuscript. All authors contributed to the article and approved the submitted version.

## Conflict of Interest

The authors declare that the research was conducted in the absence of any commercial or financial relationships that could be construed as a potential conflict of interest.

## References

[B1] Abdul Karim A. Chee P. L. Chan M. F. Loh X. J. (2016). Micellized α-cyclodextrin-based supramolecular hydrogel exhibiting pH-responsive sustained release and corresponding oscillatory shear behavior analysis. *ACS Biomater. Sci. Eng.* 2 2185–2195. 10.1021/acsbiomaterials.6b00383 33465894

[B2] Abdul Karim A. Loh X. J. (2015). Design of a micellized α-cyclodextrin based supramolecular hydrogel system. *Soft Matter* 11 5425–5434. 10.1039/c5sm00665a 26053135

[B3] Akay G. Hassan-Raeisi A. Tuncaboylu D. C. Orakdogen N. Abdurrahmanoglu S. Oppermann W. (2013). Self-healing hydrogels formed in catanionic surfactant solutions. *Soft Matter* 9 2254–2261. 10.1039/c2sm27515e

[B4] Albro M. B. Nims R. J. Durney K. M. Cigan A. D. Shim J. J. Vunjak-Novakovic G. (2016). Heterogeneous engineered cartilage growth results from gradients of media-supplemented active TGF-β and is ameliorated by the alternative supplementation of latent TGF-β. *Biomaterials* 77 173–185. 10.1016/j.biomaterials.2015.10.018 26599624 PMC4968414

[B5] Algi M. P. Okay O. (2014). Highly stretchable self-healing poly(N,N-dimethylacrylamide) hydrogels. *Eur. Polym. J.* 59 113–121. 10.1016/j.eurpolymj.2014.07.022

[B6] Amamoto Y. Otsuka H. Takahara A. Matyjaszewski K. (2012). Self-healing of covalently cross-linked polymers by reshuffling thiuram disulfide moieties in air under visible light. *Adv. Mater.* 24 3975–3980. 10.1002/adma.201201928 22730102

[B7] Appel E. A. Tibbitt M. W. Webber M. J. Mattix B. A. Veiseh O. Langer R. (2015). Self-assembled hydrogels utilizing polymer-nanoparticle interactions. *Nat. Commun.* 6:6295. 10.1038/ncomms7295 25695516 PMC4651845

[B8] Arcaute K. Mann B. K. Wicker R. B. (2006). Stereolithography of three-dimensional bioactive poly(ethylene glycol) constructs with encapsulated cells. *Ann. Biomed. Eng.* 34 1429–1441. 10.1007/s10439-006-9156-y 16897421

[B9] Baker A. E. G. Tam R. Y. Shoichet M. S. (2017). Independently tuning the biochemical and mechanical properties of 3D hyaluronan-based hydrogels with oxime and diels-alder chemistry to culture breast cancer spheroids. *Biomacromolecules* 18 4373–4384. 10.1021/acs.biomac.7b01422 29040808

[B10] Barrow S. J. Kasera S. Rowland M. J. del Barrio J. Scherman O. A. (2015). Cucurbituril-based molecular recognition. *Chem. Rev.* 115 12320–12406. 10.1021/acs.chemrev.5b00341 26566008

[B11] Benazzo F. Cadossi M. Cavani F. Fini M. Giavaresi G. Setti S. (2008). Cartilage repair with osteochondral autografts in sheep: effect of biophysical stimulation with pulsed electromagnetic fields. *J. Orthop. Res.* 26 631–642. 10.1002/jor.20530 18176941

[B12] Bert J. M. (2015). Abandoning microfracture of the knee: has the time come? *Arthroscopy* 31 501–505. 10.1016/j.arthro.2014.12.018 25744322

[B13] Bian L. Zhai D. Y. Mauck R. L. Burdick J. A. (2011). Coculture of human mesenchymal stem cells and articular chondrocytes reduces hypertrophy and enhances functional properties of engineered cartilage. *Tissue Eng. Part A* 17 1137–1145. 10.1089/ten.tea.2010.0531 21142648 PMC3063700

[B14] Bijlsma J. W. Berenbaum F. Lafeber F. P. (2011). Osteoarthritis: an update with relevance for clinical practice. *Lancet* 377 2115–2126. 10.1016/s0140-6736(11)60243-60242 21684382

[B15] Boere K. W. Visser J. Seyednejad H. Rahimian S. Gawlitta D. Van Steenbergen M. J. (2014). Covalent attachment of a three-dimensionally printed thermoplast to a gelatin hydrogel for mechanically enhanced cartilage constructs. *Acta Biomater.* 10 2602–2611. 10.1016/j.actbio.2014.02.041 24590160

[B16] Boyer C. Figueiredo L. Pace R. Lesoeur J. Rouillon T. Visage C. L. (2018). Laponite nanoparticle-associated silated hydroxypropylmethyl cellulose as an injectable reinforced interpenetrating network hydrogel for cartilage tissue engineering. *Acta Biomater.* 65 112–122. 10.1016/j.actbio.2017.11.027 29128532

[B17] Canadell J. Goossens H. Klumperman B. (2011). Self-healing materials based on disulfide links. *Macromolecules* 44 2536–2541. 10.1021/ma2001492

[B18] Castro N. J. O’Brien J. Zhang L. G. (2015). Integrating biologically inspired nanomaterials and table-top stereolithography for 3D printed biomimetic osteochondral scaffolds. *Nanoscale* 7 14010–14022. 10.1039/c5nr03425f 26234364 PMC4537413

[B19] Chang R. Nam J. Sun W. (2008). Effects of dispensing pressure and nozzle diameter on cell survival from solid freeform fabrication-based direct cell writing. *Tissue Eng. Part A* 14 41–48. 10.1089/ten.a.2007.0004 18333803

[B20] Chaudhuri O. Gu L. Darnell M. Klumpers D. Bencherif S. A. Weaver J. C. (2015). Substrate stress relaxation regulates cell spreading. *Nat. Commun.* 6:6364. 10.1038/ncomms7365 25695512 PMC4518451

[B21] Chaudhuri O. Gu L. Klumpers D. Darnell M. Bencherif S. A. Weaver J. C. (2016). Hydrogels with tunable stress relaxation regulate stem cell fate and activity. *Nat. Mater.* 15 326–334. 10.1038/nmat4489 26618884 PMC4767627

[B22] Chen G. Jiang M. (2011). Cyclodextrin-based inclusion complexation bridging supramolecular chemistry and macromolecular self-assembly. *Chem. Soc. Rev.* 40 2254–2266. 10.1039/c0cs00153h 21344115

[B23] Chen H. Sun J. Hoemann C. D. Lascau-Coman V. Ouyang W. McKee M. D. (2009). Drilling and microfracture lead to different bone structure and necrosis during bone-marrow stimulation for cartilage repair. *J. Orthop. Res.* 27 1432–1438. 10.1002/jor.20905 19402150

[B24] Chen Q. Chen H. Zhu L. Zheng J. (2015). Fundamentals of double network hydrogels. *J. Mater. Chem. B* 3 3654–3676. 32262840 10.1039/c5tb00123d

[B25] Chen T. Hou K. Ren Q. Chen G. Wei P. Zhu M. (2018). Nanoparticle-polymer synergies in nanocomposite hydrogels: from design to application. *Macromol. Rapid. Commun.* 39:e1800337. 10.1002/marc.201800337 30118163

[B26] Chirila T. V. Lee H. H. Oddon M. Nieuwenhuizen M. M. L. Nicholson T. M. (2014). Hydrogen-bonded supramolecular polymers as self-healing hydrogels: effect of a bulky adamantyl substituent in the ureido-pyrimidinone monomer. *J. Appl. Polym.* 131, 1001–1007. 10.1002/app.39932

[B27] Chung J. H. Y. Naficy S. Yue Z. Kapsa R. Quigley A. Moulton S. E. (2013). Bio-ink properties and printability for extrusion printing living cells. *Biomater. Sci.* 1 763–773. 10.1039/c3bm00012e 32481829

[B28] Dai X. Zhang Y. Gao L. Bai T. Wang W. Cui Y. (2015). A mechanically strong, highly stable, thermoplastic, and self-healable supramolecular polymer hydrogel. *Adv. Mater.* 27 3566–3571. 10.1002/adma.201500534 25946310

[B29] Daly A. C. Critchley S. E. Rencsok E. M. Kelly D. J. (2016). A comparison of different bioinks for 3D bioprinting of fibrocartilage and hyaline cartilage. *Biofabrication* 8:045002 10.1088/1758-5090/8/4/045002 27716628

[B30] Dankers P. Y. Hermans T. M. Baughman T. W. Kamikawa Y. Kieltyka R. E. Bastings M. M. (2012). Hierarchical formation of supramolecular transient networks in water: a modular injectable delivery system. *Adv. Mater.* 24 2703–2709. 10.1002/adma.201104072 22528786

[B31] Dawson J. I. Kanczler J. M. Yang X. B. Attard G. S. Oreffo R. O. (2011). Clay gels for the delivery of regenerative microenvironments. *Adv. Mater.* 23 3304–3308. 10.1002/adma.201100968 21661063

[B32] Deng G. Li F. Yu H. Liu F. Liu C. Sun W. (2012). Dynamic hydrogels with an environmental adaptive self-healing ability and dual responsive sol-gel transitions. *ACS Macro Lett. Lett.* 1 275–279. 10.1021/mz200195n 35578522

[B33] Ding H. Zhang X. N. Zheng S. Y. Song Y. Wu Z. L. Zheng Q. (2017). Hydrogen bond reinforced poly (1-vinylimidazole-co-acrylic acid) hydrogels with high toughness, fast self-recovery, and dual pH-responsiveness. *Polymer* 131 95–103. 10.1016/j.polymer.2017.09.044

[B34] Dowlut M. Hall D. G. (2006). An improved class of sugar-binding boronic acids, soluble and capable of complexing glycosides in neutral water. *J. Am. Chem. Soc.* 128 4226–4227. 10.1021/ja057798c 16568987

[B35] Engel A. K. Yoden T. Sanui K. Ogata N. (1985). Synthesis of aromatic Schiff base oligomers at the air/water interface. *J. Am. Chem. Soc.* 107 8308–8310. 10.1021/ja00312a108

[B36] Fellows C. R. Matta C. Zakany R. Khan I. M. Mobasheri A. (2016). Adipose, bone marrow and synovial joint-derived mesenchymal stem cells for cartilage repair. *Front. Genet.* 7:213. 10.3389/fgene.2016.00213 28066501 PMC5167763

[B37] Feng Q. Wei K. Lin S. Xu Z. Sun Y. Shi P. (2016). Mechanically resilient, injectable, and bioadhesive supramolecular gelatin hydrogels crosslinked by weak host-guest interactions assist cell infiltration and in situ tissue regeneration. *Biomaterials* 101 217–228. 10.1016/j.biomaterials.2016.05.043 27294539

[B38] Gaharwar A. K. Peppas N. A. Khademhosseini A. (2014). Nanocomposite hydrogels for biomedical applications. *Biotechnol. Bioeng.* 111 441–453. 10.1002/bit.25160 24264728 PMC3924876

[B39] Gaharwar A. K. Schexnailder P. J. Kline B. P. Schmidt G. (2011). Assessment of using laponite cross-linked poly(ethylene oxide) for controlled cell adhesion and mineralization. *Acta Biomater.* 7 568–577. 10.1016/j.actbio.2010.09.015 20854941

[B40] Galperin A. Oldinski R. A. Florczyk S. J. Bryers J. D. Zhang M. Ratner B. D. (2013). Integrated bi-layered scaffold for osteochondral tissue engineering. *Adv. Healthc. Mater.* 2 872–883. 10.1002/adhm.201200345 23225568 PMC3644393

[B41] Gao F. Xu Z. Liang Q. Li H. Peng L. Wu M. (2019). Osteochondral regeneration with 3D-printed biodegradable high-strength supramolecular polymer reinforced-gelatin hydrogel scaffolds. *Adv. Sci.* 6:1900867. 10.1002/advs.201900867 31406678 PMC6685475

[B42] Gemert G. M. L. V. Peeters J. W. Sntjens S. H. M. Janssen H. M. Bosman A. W. (2012). Self–Healing supramolecular polymers in action. *Macromol. Chem. Phys.* 213 234–242.

[B43] Giannoni P. Pagano A. Maggi E. Arbicò R. Randazzo N. Grandizio M. (2005). Autologous chondrocyte implantation (ACI) for aged patients: development of the proper cell expansion conditions for possible therapeutic applications. *Osteoarthr. Cartil.* 13 589–600. 10.1016/j.joca.2005.02.015 15979011

[B44] Gong J. P. (2010). Why are double network hydrogels so tough? *Soft Matter* 6 2583–2590. 10.1039/b924290b

[B45] Gopinathan J. Noh I. (2018). Recent trends in bioinks for 3D printing. *Biomater. Res.* 22:11 10.1186/s40824-018-0122-121 PMC5889544 29636985

[B46] Gou G. H. Tseng F. J. Wang S. H. Chen P. J. Shyu J. F. Weng C. F. (2020). Autologous chondrocyte implantation versus microfracture in the knee: a meta-analysis and systematic review. *Arthroscopy* 36 289–303. 10.1016/j.arthro.2019.06.033 31708355

[B47] Grindy S. C. Learsch R. Mozhdehi D. Cheng J. Barrett D. G. Guan Z. (2015). Control of hierarchical polymer mechanics with bioinspired metal-coordination dynamics. *Nat. Mater.* 14 1210–1216. 10.1038/nmat4401 26322715 PMC4654658

[B48] Gulyuz U. Okay O. (2014). Self-healing poly(acrylic acid) hydrogels with shape memory behavior of high mechanical strength. *Macromolecules* 47 6889–6899. 10.1021/ma5015116

[B49] Guo Z. Ma W. Gu H. Feng Y. He Z. Chen Q. (2017). pH-Switchable and self-healable hydrogels based on ketone type acylhydrazone dynamic covalent bonds. *Soft Matter* 13 7371–7380. 10.1039/c7sm00916j 28951902

[B50] Haene R. Qamirani E. Story R. A. Pinsker E. Daniels T. R. (2012). Intermediate outcomes of fresh talar osteochondral allografts for treatment of large osteochondral lesions of the talus. *J. Bone Joint. Surg. Am.* 94 1105–1110. 10.2106/jbjs.j.02010 22717829

[B51] Haldar U. Bauri K. Li R. Faust R. De P. (2015). Polyisobutylene-Based pH-responsive self-healing polymeric gels. *ACS Appl. Mater. Interf.* 7 8779–8788. 10.1021/acsami.5b01272 25844579

[B52] Harada A. Li J. Kamachi M. (1992). The molecular necklace: a rotaxane containing many threaded α-cyclodextrins. *Nature* 356 325–327. 10.1038/356325a0

[B53] Harris J. D. Siston R. A. Pan X. Flanigan D. C. (2010). Autologous chondrocyte implantation: a systematic review. *J. Bone Joint. Surg. Am.* 92 2220–2233. 10.2106/jbjs.j.00049 20844166 PMC7373451

[B54] He L. Fullenkamp D. E. Rivera J. G. Messersmith P. B. (2011). pH responsive self-healing hydrogels formed by boronate-catechol complexation. *Chem. Commun.* 47 7497–7499. 10.1039/c1cc11928a 21629956 PMC4526106

[B55] Hendriks J. Riesle J. van Blitterswijk C. A. (2007). Co-culture in cartilage tissue engineering. *J. Tissue Eng. Regener. Med.* 1 170–178. 10.1002/term.19 18038408

[B56] Highley C. B. Rodell C. B. Burdick J. A. (2015). Direct 3D printing of shear-thinning hydrogels into self-healing hydrogels. *Adv. Mater.* 27 5075–5079. 10.1002/adma.201501234 26177925

[B57] Hölzl K. Lin S. Tytgat L. Van Vlierberghe S. Gu L. Ovsianikov A. (2016). Bioink properties before, during and after 3D bioprinting. *Biofabrication* 8:032002 10.1088/1758-5090/8/3/032002 27658612

[B58] Hong S. Sycks D. Chan H. F. Lin S. Lopez G. P. Guilak F. (2015a). 3D printing of highly stretchable and tough hydrogels into complex, cellularized structures. *Adv. Mater.* 27 4035–4040. 10.1002/adma.201501099 26033288 PMC4849481

[B59] Hong S. Sycks D. Chan H. F. Lin S. Lopez G. P. Guilak F. (2015b). 3D printing: 3D printing of highly stretchable and tough hydrogels into complex, cellularized structures. *Adv. Mater.* 27:4034. 10.1002/adma.201570182 26172844

[B60] Hu X. Vatankhah-Varnoosfaderani M. Zhou J. Li Q. Sheiko S. S. (2015). Weak hydrogen bonding enables hard, strong, tough, and elastic hydrogels. *Adv. Mater.* 27 6899–6905. 10.1002/adma.201503724 26436409

[B61] Huey D. J. Hu J. C. Athanasiou K. A. (2012). Unlike bone, cartilage regeneration remains elusive. *Science* 338 917–921. 10.1126/science.1222454 23161992 PMC4327988

[B62] Jayaraman P. Gandhimathi C. Venugopal J. R. Becker D. L. Ramakrishna S. Srinivasan D. K. (2015). Controlled release of drugs in electrosprayed nanoparticles for bone tissue engineering. *Adv. Drug Deliv. Rev.* 94 77–95. 10.1016/j.addr.2015.09.007 26415888

[B63] Jeon O. Alt D. S. Linderman S. W. Alsberg E. (2013). Biochemical and physical signal gradients in hydrogels to control stem cell behavior. *Adv. Mater.* 25 6366–6372. 10.1002/adma.201302364 23983019 PMC3863582

[B64] Jiang G. Liu C. Liu X. Zhang G. Yang M. Chen Q. (2010). Self-healing mechanism and mechanical behavior of hydrophobic association hydrogels with high mechanical strength. *J. Macromol. Sci. Part A Pure Appl. Chem.* 47 335–342. 10.1080/10601320903539272

[B65] Kai D. Low Z. W. Liow S. S. Abdul Karim A. Ye H. Jin G. (2015). Development of lignin supramolecular hydrogels with mechanically responsive and self-healing properties. *ACS Sustain. Chem. Eng.* 3 2160–2169. 10.1021/acssuschemeng.5b00405

[B66] Kieltyka R. E. Pape A. C. Albertazzi L. Nakano Y. Bastings M. M. Voets I. K. (2013). Mesoscale modulation of supramolecular ureidopyrimidinone-based poly(ethylene glycol) transient networks in water. *J. Am. Chem. Soc.* 135 11159–11164. 10.1021/ja403745w 23829684

[B67] Leijten J. C. Georgi N. Wu L. van Blitterswijk C. A. Karperien M. (2013). Cell sources for articular cartilage repair strategies: shifting from monocultures to cocultures. *Tissue Eng. Part B Rev.* 19 31–40. 10.1089/ten.teb.2012.0273 22845048

[B68] Leppiniemi J. Lahtinen P. Paajanen A. Mahlberg R. Metsä-Kortelainen S. Pinomaa T. (2017). 3D-printable bioactivated nanocellulose-alginate hydrogels. *ACS Appl. Mater. Interf.* 9 21959–21970. 10.1021/acsami.7b02756 28598154

[B69] Li C. H. Wang C. Keplinger C. Zuo J. L. Jin L. Sun Y. (2016). A highly stretchable autonomous self-healing elastomer. *Nat. Chem.* 8 618–624. 10.1038/nchem.2492 27219708

[B70] Li H. Tan Y. J. Leong K. F. Li L. (2017). 3D bioprinting of highly thixotropic alginate/methylcellulose hydrogel with strong interface bonding. *ACS Appl. Mater. Interf.* 9 20086–20097. 10.1021/acsami.7b04216 28530091

[B71] Li J. Celiz A. D. Yang J. Yang Q. Wamala I. Whyte W. (2017). Tough adhesives for diverse wet surfaces. *Science* 357 378–381. 10.1126/science.aah6362 28751604 PMC5905340

[B72] Li S. Wang J. Song L. Zhou Y. Zhao J. Hou X. (2016). Injectable PAMAM/ODex double-crosslinked hydrogels with high mechanical strength. *Biomed. Mater.* 12:015012. 10.1088/1748-605x/12/1/015012 27934783

[B73] Liao X. Chen G. Liu X. Chen W. Chen F. Jiang M. (2010). Photoresponsive pseudopolyrotaxane hydrogels based on competition of host-guest interactions. *Angew. Chem. Int. Edn. Engl.* 49 4409–4413. 10.1002/anie.201000141 20480467

[B74] Lin K. F. He S. Song Y. Wang C. M. Gao Y. Li J. Q. (2016). Low-temperature additive manufacturing of biomimic three-dimensional Hydroxyapatite/collagen scaffolds for bone regeneration. *ACS Appl. Mater. Interf.* 8 6905–6916. 10.1021/acsami.6b00815 26930140

[B75] Liow S. S. Zhou H. Sugiarto S. Guo S. Chalasani M. L. S. Verma N. K. (2017). Highly efficient supramolecular aggregation-induced emission-active pseudorotaxane luminogen for functional bioimaging. *Biomacromolecules* 18 886–897. 10.1021/acs.biomac.6b01777 28140561

[B76] Liu J. Tan C. S. Yu Z. Lan Y. Abell C. Scherman O. A. (2017a). Biomimetic supramolecular polymer networks exhibiting both toughness and self-recovery. *Adv. Mater.* 29:1604951. 10.1002/adma.201604951 28092128

[B77] Liu J. Tan C. S. Y. Yu Z. Li N. Abell C. Scherman O. A. (2017b). Tough supramolecular polymer networks with extreme stretchability and fast room-temperature self-healing. *Adv. Mater.* 29:1605325. 10.1002/adma.201605325 28370560

[B78] Liu M. Zeng X. Ma C. Yi H. Ali Z. Mou X. (2017c). Injectable hydrogels for cartilage and bone tissue engineering. *Bone Res.* 5:17014. 10.1038/boneres.2017.14 28584674 PMC5448314

[B79] Liu S. Pu Y. Yang R. Liu X. Wang P. Wang X. (2020). Boron-assisted dual-crosslinked poly (γ-glutamic acid) hydrogels with high toughness for cartilage regeneration. *Int. J. Biol. Macromol.* 153 158–168. 10.1016/j.ijbiomac.2020.02.314 32114174

[B80] Liu X. Yang Y. Li Y. Niu X. Zhao B. Wang Y. (2017d). Integration of stem cell-derived exosomes with in situ hydrogel glue as a promising tissue patch for articular cartilage regeneration. *Nanoscale* 9 4430–4438. 10.1039/c7nr00352h 28300264

[B81] Liu X. Yang Y. Niu X. Lin Q. Zhao B. Wang Y. (2017e). An in situ photocrosslinkable platelet rich plasma - complexed hydrogel glue with growth factor controlled release ability to promote cartilage defect repair. *Acta Biomater.* 62 179–187. 10.1016/j.actbio.2017.05.023 28501713

[B82] Liu Y. L. Chuo T. W. (2013). Self-healing polymers based on thermally reversible Diels-Alder chemistry. *Polym. Chem.* 4:2194 10.1039/c2py20957h

[B83] Loebel C. Rodell C. B. Chen M. H. Burdick J. A. (2017). Shear-thinning and self-healing hydrogels as injectable therapeutics and for 3D-printing. *Nat. Protoc.* 12 1521–1541. 10.1038/nprot.2017.053 28683063 PMC7546336

[B84] MacDonald A. E. Bedi A. Horner N. S. de Sa D. Simunovic N. Philippon M. J. (2016). Indications and outcomes for microfracture as an adjunct to hip arthroscopy for treatment of chondral defects in patients with femoroacetabular impingement: a systematic review. *Arthroscopy* 32 190–200.e192. 10.1016/j.arthro.2015.06.041 26385287

[B85] Mann J. L. Yu A. C. Agmon G. Appel E. A. (2017). Supramolecular polymeric biomaterials. *Biomater. Sci.* 6 10–37. 10.1039/c7bm00780a 29164196

[B86] McConnell A. J. Wood C. S. Neelakandan P. P. Nitschke J. R. (2015). Stimuli-responsive metal-ligand assemblies. *Chem. Rev.* 115 7729–7793. 10.1021/cr500632f 25880789

[B87] Means A. K. Shrode C. S. Whitney L. V. Ehrhardt D. A. Grunlan M. A. (2019). Double network hydrogels that mimic the modulus, strength, and lubricity of cartilage. *Biomacromolecules* 20 2034–2042. 10.1021/acs.biomac.9b00237 31009565

[B88] Menyo M. S. Hawker C. J. Waite J. H. (2015). Rate-dependent stiffness and recovery in interpenetrating network hydrogels through sacrificial metal coordination bonds. *ACS Macro Lett.* 4 1200–1204. 10.1021/acsmacrolett.5b00664 27818845 PMC5096649

[B89] Moeinzadeh S. Shariati S. R. P. Jabbari E. (2016). Comparative effect of physicomechanical and biomolecular cues on zone-specific chondrogenic differentiation of mesenchymal stem cells. *Biomaterials* 92 57–70. 10.1016/j.biomaterials.2016.03.034 27038568 PMC4833585

[B90] Mozhdehi D. Neal J. A. Grindy S. C. Cordeau Y. Ayala S. Holten-Andersen N. (2016). Tuning dynamic mechanical response in metallopolymer networks through simultaneous control of structural and temporal properties of the networks. *Macromolecules* 49 6310–6321. 10.1021/acs.macromol.6b01626

[B91] Nair K. Gandhi M. Khalil S. Yan K. C. Marcolongo M. Barbee K. (2009). Characterization of cell viability during bioprinting processes. *Biotechnol. J.* 4 1168–1177. 10.1002/biot.200900004 19507149

[B92] Ning L. Chen X. (2017). A brief review of extrusion-based tissue scaffold bio-printing. *Biotechnol. J.* 12:1600671. 10.1002/biot.201600671 28544779

[B93] Otto S. Engberts J. B. (2003). Hydrophobic interactions and chemical reactivity. *Org. Biomol. Chem.* 1 2809–2820. 10.1039/b305672d 12968330

[B94] Ouyang L. Yao R. Zhao Y. Sun W. (2016). Effect of bioink properties on printability and cell viability for 3D bioplotting of embryonic stem cells. *Biofabrication* 8:035020 10.1088/1758-5090/8/3/035020 27634915

[B95] Ozbolat I. T. Hospodiuk M. (2016). Current advances and future perspectives in extrusion-based bioprinting. *Biomaterials* 76 321–343. 10.1016/j.biomaterials.2015.10.076 26561931

[B96] Palaganas N. B. Mangadlao J. D. de Leon A. C. C. Palaganas J. O. Pangilinan K. D. Lee Y. J. (2017). 3D printing of photocurable cellulose nanocrystal composite for fabrication of complex architectures via stereolithography. *ACS Appl. Mater. Interf.* 9 34314–34324. 10.1021/acsami.7b09223 28876895

[B97] Pascual-Garrido C. Rodriguez-Fontan F. Aisenbrey E. A. Payne K. A. Chahla J. Goodrich L. R. (2018). Current and novel injectable hydrogels to treat focal chondral lesions: properties and applicability. *J. Orthop. Res.* 36 64–75. 10.1002/jor.23760 28975658 PMC5839960

[B98] Paul A. Manoharan V. Krafft D. Assmann A. Uquillas J. A. Shin S. R. (2016). Nanoengineered biomimetic hydrogels for guiding human stem cell osteogenesis in three dimensional microenvironments. *J. Mater. Chem. B* 4 3544–3554. 10.1039/c5tb02745d 27525102 PMC4980085

[B99] Pelgrift R. Y. Friedman A. J. (2013). Nanotechnology as a therapeutic tool to combat microbial resistance. *Adv. Drug Deliv. Rev.* 65 1803–1815. 10.1016/j.addr.2013.07.011 23892192

[B100] Polat G. Erşen A. Erdil M. E. Kızılkurt T. Kılıçoğlu Ö Aşık M. (2016). Long-term results of microfracture in the treatment of talus osteochondral lesions. *Knee Surg. Sports Traumatol. Arthrosc.* 24 1299–1303. 10.1007/s00167-016-3990-3998 26831855

[B101] Qu D.-H. Wang Q.-C. Zhang Q.-W. Ma X. Tian H. (2015). Photoresponsive host-guest functional systems. *Chem. Rev.* 115 7543–7588. 10.1021/cr5006342 25697681

[B102] Radhakrishnan J. Manigandan A. Chinnaswamy P. Subramanian A. Sethuraman S. (2018). Gradient nano-engineered in situ forming composite hydrogel for osteochondral regeneration. *Biomaterials* 162 82–98. 10.1016/j.biomaterials.2018.01.056 29438883

[B103] Rastogi P. Kandasubramanian B. (2019). Review of alginate-based hydrogel bioprinting for application in tissue engineering. *Biofabrication* 11:042001. 10.1088/1758-5090/ab331e 31315105

[B104] Rausch Osthoff A. K. Niedermann K. Braun J. Adams J. Brodin N. Dagfinrud H. (2018). 2018 EULAR recommendations for physical activity in people with inflammatory arthritis and osteoarthritis. *Ann. Rheum. Dis.* 77 1251–1260. 10.1136/annrheumdis-2018-213585 29997112

[B105] Redondo M. L. Beer A. J. Yanke A. B. (2018). Cartilage restoration: microfracture and osteochondral autograft transplantation. *J. Knee Surg.* 31 231–238. 10.1055/s-0037-1618592 29396963

[B106] Ribeiro V. P. Pina S. Oliveira J. M. Reis R. L. (2018). Silk fibroin-based hydrogels and scaffolds for osteochondral repair and regeneration. *Adv. Exp. Med. Biol.* 1058 305–325. 10.1007/978-3-319-76711-6_14 29691828

[B107] Richardson S. M. Kalamegam G. Pushparaj P. N. Matta C. Memic A. Khademhosseini A. (2016). Mesenchymal stem cells in regenerative medicine: focus on articular cartilage and intervertebral disc regeneration. *Methods* 99 69–80. 10.1016/j.ymeth.2015.09.015 26384579

[B108] Roberts M. C. Hanson M. C. Massey A. P. Karren E. A. Kiser P. F. (2010). Dynamically restructuring hydrogel networks formed with reversible covalent crosslinks. *Adv. Mater.* 19 2503–2507. 10.1002/adma.200602649

[B109] Rodell C. B. Dusaj N. N. Highley C. B. Burdick J. A. (2016). Injectable and cytocompatible tough double-network hydrogels through tandem supramolecular and covalent crosslinking. *Adv. Mater.* 28 8419–8424. 10.1002/adma.201602268 27479881 PMC7437955

[B110] Roos E. M. Arden N. K. (2016). Strategies for the prevention of knee osteoarthritis. *Nat. Rev. Rheumatol.* 12 92–101. 10.1038/nrrheum.2015.135 26439406

[B111] Sahoo J. K. VandenBerg M. A. Webber M. J. (2018). Injectable network biomaterials via molecular or colloidal self-assembly. *Adv. Drug Deliv. Rev.* 127 185–207. 10.1016/j.addr.2017.11.005 29128515

[B112] Schlichting K. E. Copeland-Johnson T. M. Goodman M. Lipert R. J. Prozorov T. Liu X. (2011). Synthesis of a novel photopolymerized nanocomposite hydrogel for treatment of acute mechanical damage to cartilage. *Acta Biomater.* 7 3094–3100. 10.1016/j.actbio.2011.04.010 21530694 PMC4950507

[B113] Schuurman W. Harimulyo E. Gawlitta D. Woodfield T. Dhert W. J. van Weeren P. R. (2016). Three-dimensional assembly of tissue-engineered cartilage constructs results in cartilaginous tissue formation without retainment of zonal characteristics. *J. Tissue Eng. Regener. Med.* 10 315–324. 10.1002/term.1726 23606563

[B114] Selmi T. A. Verdonk P. Chambat P. Dubrana F. Potel J. F. Barnouin L. (2008). Autologous chondrocyte implantation in a novel alginate-agarose hydrogel: outcome at two years. *J. Bone Joint. Surg. Br.* 90 597–604. 10.1302/0301-620x.90b5.20360 18450625

[B115] Shin S. R. Jung S. M. Zalabany M. Kim K. Zorlutuna P. Kim S. B. (2013). Carbon-nanotube-embedded hydrogel sheets for engineering cardiac constructs and bioactuators. *ACS Nano* 7 2369–2380. 10.1021/nn305559j 23363247 PMC3609875

[B116] Skardal A. Zhang J. McCoard L. Oottamasathien S. Prestwich G. D. (2010). Dynamically crosslinked gold nanoparticle - hyaluronan hydrogels. *Adv. Mater.* 22 4736–4740. 10.1002/adma.201001436 20730818

[B117] Sun J. Y. Zhao X. Illeperuma W. R. Chaudhuri O. Oh K. H. Mooney D. J. (2012). Highly stretchable and tough hydrogels. *Nature* 489 133–136. 10.1038/nature11409 22955625 PMC3642868

[B118] Thi P. L. Lee Y. Tran D. L. Thi T. T. H. Kang J. I. Park K. M. (2020). In situ forming and reactive oxygen species-scavenging gelatin hydrogels for enhancing wound healing efficacy. *Acta Biomater.* 103 142–152. 10.1016/j.actbio.2019.12.009 31846801

[B119] Tibbitt M. W. Anseth K. S. (2009). Hydrogels as extracellular matrix mimics for 3D cell culture. *Biotechnol. Bioeng.* 103 655–663. 10.1002/bit.22361 19472329 PMC2997742

[B120] Tuan R. S. (2007). A second-generation autologous chondrocyte implantation approach to the treatment of focal articular cartilage defects. *Arthrit. Res. Ther.* 9:109. 10.1186/ar2310 18021426 PMC2212558

[B121] Tuncaboylu D. C. Argun A. Algi M. P. Okay O. (2013). Autonomic self-healing in covalently crosslinked hydrogels containing hydrophobic domains. *Polymer* 54 6381–6388. 10.1016/j.polymer.2013.09.051

[B122] Tuncaboylu D. C. Sari M. Oppermann W. Okay O. (2011). Tough and Self-healing hydrogels formed via hydrophobic interactions. *Macromolecules* 44 4997–5005. 10.1021/ma200579v

[B123] Van Hoorick J. Tytgat L. Dobos A. Ottevaere H. Van Erps J. Thienpont H. (2019). (Photo-)crosslinkable gelatin derivatives for biofabrication applications. *Acta Biomater.* 97 46–73. 10.1016/j.actbio.2019.07.035 31344513

[B124] Vega S. L. Kwon M. Y. Burdick J. A. (2017). Recent advances in hydrogels for cartilage tissue engineering. *Eur. Cell Mater.* 33 59–75. 10.22203/eCM.v033a05 28138955 PMC5748291

[B125] Visser J. Melchels F. P. Jeon J. E. Van Bussel E. M. Kimpton L. S. Byrne H. M. (2015). Reinforcement of hydrogels using three-dimensionally printed microfibres. *Nat. Commun.* 6 1–10. 10.1038/ncomms7933 25917746

[B126] Wang J. Li Q. Yi S. Chen X. (2017). Visible-light/temperature dual-responsive hydrogel constructed by α-cyclodextrin and an azobenzene linked surfactant. *Soft Matter* 13 6490–6498. 10.1039/c7sm01528c 28880328

[B127] Wang W. Zhang Y. Liu W. (2017). Bioinspired fabrication of high strength hydrogels from non-covalent interactions. *Prog. Polym. Sci.* 71 1–25. 10.1016/j.progpolymsci.2017.04.001

[B128] Wang L. L. Highley C. B. Yeh Y. C. Galarraga J. H. Uman S. Burdick J. A. (2018). Three-dimensional extrusion bioprinting of single- and double-network hydrogels containing dynamic covalent crosslinks. *J. Biomed. Mater. Res. A* 106 865–875. 10.1002/jbm.a.36323 29314616 PMC5826872

[B129] Wang X. Wenk E. Zhang X. Meinel L. Vunjak-Novakovic G. Kaplan D. L. (2009). Growth factor gradients via microsphere delivery in biopolymer scaffolds for osteochondral tissue engineering. *J. Control. Release* 134 81–90. 10.1016/j.jconrel.2008.10.021 19071168 PMC2698962

[B130] Wang Z. An G. Zhu Y. Liu X. Chen Y. Wu H. (2019). 3D-printable self-healing and mechanically reinforced hydrogels with host-guest non-covalent interactions integrated into covalently linked networks. *Mater. Horiz.* 6 733–742. 10.1039/c8mh01208c 31572613 PMC6768557

[B131] Wei K. Zhu M. Sun Y. Xu J. Feng Q. Lin S. (2016). Robust biopolymeric supramolecular “Host–Guest Macromer” hydrogels reinforced by in situ formed multivalent nanoclusters for cartilage regeneration. *Macromolecules* 49 866–875. 10.1021/acs.macromol.5b02527

[B132] Winnik M. A. Yekta A. (1997). Associative polymers in aqueous solution. *Curr. Opin. Colloid Interf. Sci.* 2 424–436. 10.1016/S1359-0294(97)80088-X

[B133] Winter A. Schubert U. S. (2016). Synthesis and characterization of metallo-supramolecular polymers. *Chem. Soc. Rev.* 45 5311–5357. 10.1039/c6cs00182c 27218823

[B134] Xavier J. R. Thakur T. Desai P. Jaiswal M. K. Sears N. Cosgriff-Hernandez E. (2015). Bioactive nanoengineered hydrogels for bone tissue engineering: a growth-factor-free approach. *ACS Nano* 9 3109–3118. 10.1021/nn507488s 25674809

[B135] Xu C. Lee W. Dai G. Hong Y. (2018). Highly elastic biodegradable single-network hydrogel for cell printing. *ACS Appl. Mater. Interf.* 10 9969–9979. 10.1021/acsami.8b01294 29451384 PMC5876623

[B136] Xu Y. Li Y. Chen Q. Fu L. Tao L. Wei Y. (2018). Injectable and self-healing chitosan hydrogel based on imine bonds: design and therapeutic applications. *Int. J. Mol. Sci.* 19:2198. 10.3390/ijms19082198 30060504 PMC6121669

[B137] Xu J. Feng Q. Lin S. Yuan W. Li R. Li J. (2019). Injectable stem cell-laden supramolecular hydrogels enhance in situ osteochondral regeneration via the sustained co-delivery of hydrophilic and hydrophobic chondrogenic molecules. *Biomaterials* 210 51–61. 10.1016/j.biomaterials.2019.04.031 31075723

[B138] Xue K. Liow S. S. Karim A. A. Li Z. Loh X. J. (2018). A recent perspective on noncovalently formed polymeric hydrogels. *Chem. Rec.* 18 1517–1529. 10.1002/tcr.201800015 29791779

[B139] Yamaguchi H. Kobayashi Y. Kobayashi R. Takashima Y. Hashidzume A. Harada A. (2012). Photoswitchable gel assembly based on molecular recognition. *Nat. Commun.* 3:603. 10.1038/ncomms1617 22215078 PMC3272571

[B140] Ye H. Owh C. Loh X. J. (2015). A thixotropic polyglycerol sebacate-based supramolecular hydrogel showing UCST(behavior). *RSC Adv.* 5 48720–48728. 10.1039/c5ra08222f PMC6432133 30979218

[B141] Yesilyurt V. Webber M. J. Appel E. A. Godwin C. Langer R. Anderson D. G. (2016). Injectable self-healing glucose-responsive hydrogels with pH-regulated mechanical properties. *Adv. Mater.* 28 86–91. 10.1002/adma.201502902 26540021 PMC4825176

[B142] Ying H. Zhang Y. Cheng J. (2014). Dynamic urea bond for the design of reversible and self-healing polymers. *Nat. Commun.* 5:3218. 10.1038/ncomms4218 24492620 PMC4438999

[B143] Yu F. Cao X. Du J. Wang G. Chen X. (2015). Multifunctional hydrogel with good structure integrity, self-healing, and tissue-adhesive property formed by combining diels-alder click reaction and acylhydrazone bond. *ACS Appl. Mater. Interf.* 7 24023–24031. 10.1021/acsami.5b06896 26466997

[B144] Zhai X. Ma Y. Hou C. Gao F. Zhang Y. Ruan C. (2017). 3D-printed high strength bioactive supramolecular polymer/clay nanocomposite hydrogel scaffold for bone regeneration. *ACS Biomater. Sci. Eng.* 3 1109–1118. 10.1021/acsbiomaterials.7b00224 33429585

[B145] Zhang J. Yang Y. Chen Y. Liu X. Guo S. Zhu L. (2016). An in situ phototriggered-imine-crosslink composite hydrogel for bone defect repair. *J. Mater. Chem. B* 4 973–981. 10.1039/c5tb02377g 32263170

[B146] Zhang N. Lock J. Sallee A. Liu H. (2015). Magnetic nanocomposite hydrogel for potential cartilage tissue engineering: synthesis, characterization, and cytocompatibility with bone marrow derived mesenchymal stem cells. *ACS Appl. Mater. Interf.* 7:20987. 10.1021/acsami.5b06939 26360342

[B147] Zhang X. N. Wang Y. J. Sun S. Hou L. Wu P. Wu Z. L. (2018). A tough and stiff hydrogel with tunable water content and mechanical properties based on the synergistic effect of hydrogen bonding and hydrophobic interaction. *Macromolecules* 51 8136–8146. 10.1021/acs.macromol.8b01496

[B148] Zhang Y. Tao L. Li S. Wei Y. (2011). Synthesis of multiresponsive and dynamic chitosan-based hydrogels for controlled release of bioactive molecules. *Biomacromolecules* 12 2894–2901. 10.1021/bm200423f 21699141

[B149] Zhao Y. Li M. Liu B. Xiang J. Cui Z. Qu X. (2018). Ultra-tough injectable cytocompatible hydrogel for 3D cell culture and cartilage repair. *J. Mater. Chem. B* 6 1351–1358. 10.1039/c7tb03177g 32254420

[B150] Zhao Y. Li Y. Mao S. Sun W. Yao R. (2015). The influence of printing parameters on cell survival rate and printability in microextrusion-based 3D cell printing technology. *Biofabrication* 7:045002 10.1088/1758-5090/7/4/045002 26523399

[B151] Zhao Y. L. Stoddart J. F. (2009). Azobenzene-based light-responsive hydrogel system. *Langmuir* 25 8442–8446. 10.1021/la804316u 20050041

[B152] Zheng L. Jiang X. Chen X. Fan H. Zhang X. (2014). Evaluation of novel in situ synthesized nano-hydroxyapatite/collagen/alginate hydrogels for osteochondral tissue engineering. *Biomed. Mater.* 9:065004 10.1088/1748-6041/9/6/065004 25358331

[B153] Zhu D. Wang H. Trinh P. Heilshorn S. C. Yang F. (2017). Elastin-like protein-hyaluronic acid (ELP-HA) hydrogels with decoupled mechanical and biochemical cues for cartilage regeneration. *Biomaterials* 127 132–140. 10.1016/j.biomaterials.2017.02.010 28268018 PMC5772736

